# Neonatal ketone body elevation regulates postnatal heart development by promoting cardiomyocyte mitochondrial maturation and metabolic reprogramming

**DOI:** 10.1038/s41421-022-00447-6

**Published:** 2022-10-11

**Authors:** Danyang Chong, Yayun Gu, Tongyu Zhang, Yu Xu, Dandan Bu, Zhong Chen, Na Xu, Liangkui Li, Xiyu Zhu, Haiquan Wang, Yangqing Li, Feng Zheng, Dongjin Wang, Peng Li, Li Xu, Zhibin Hu, Chaojun Li

**Affiliations:** 1 Gusu School, State Key Laboratory of Reproductive Medicine and China International Joint Research Center on Environment and Human Health, Center for Global Health, School of Public Health, Nanjing Medical University, Nanjing, Jiangsu China; 2 Model Animal Research Centre (MARC), Medical School of Nanjing University, National Resource Centre for Mutant Mice, Nanjing, Jiangsu China; 3 State Key Laboratory of Membrane Biology and Tsinghua-Peking Center for Life Sciences, School of Life Sciences, Tsinghua University, Beijing, China; 4 Department of Cardio-Thoracic Surgery, Nanjing Drum Tower Hospital, The Affiliated Hospital of Nanjing University Medical School, Nanjing, Jiangsu China

**Keywords:** Reprogramming, Acetylation

## Abstract

Neonatal heart undergoes metabolic conversion and cell cycle arrest preparing for the increased workload during adulthood. Herein, we report that neonatal ketone body elevation is a critical regulatory factor for postnatal heart development. Through multiomics screening, we found that the expression of 3-hydroxy-3-methylglutaryl-CoA synthase 2 (HMGCS2), the rate-limiting enzyme of ketogenesis, was transiently induced by colostrum in the neonatal heart. *Hmgcs2* knockout caused mitochondrial maturation defects. Meanwhile, postnatal heart development was compromised and cardiomyocytes reacquired proliferation capacity in *Hmgcs2* knockout mice. Consequently, over 40% of newborn *Hmgcs2* knockout mice died before weaning. The heart function of surviving *Hmgcs2* knockout mice was also impaired, which could be rescued by ketone body supplementation during the suckling stage. Mechanistically, ketone body deficiency inhibited β-hydroxybutyrylation but enhanced acetylation of mitochondrial proteins, which might be responsible for the inhibition of the enzyme activity in mitochondria. These observations suggest that ketone body is critical for postnatal heart development through regulating mitochondrial maturation and metabolic reprogramming.

## Introduction

The cells of various organs in newborns must undergo a series of reprogramming processes to adapt to the postnatal environment. It is not only external factors such as temperature and oxygen that change dramatically after birth, factors of the internal milieu, such as metabolic substrates, are also largely altered after birth. For example, transplacental supplementation with carbohydrates, primarily glucose and lactate, provides energy for myocardial contraction in the fetal heart^1^. The acquisition of dietary lipids from colostrum provokes a metabolic shift from anaerobic glycolysis to aerobic fatty acid (FA) beta-oxidation in mitochondria since spontaneous breathing allows the newborn to access more oxygen^2–4^. This metabolic reprogramming occurs in just a few days after birth.

Along with metabolic reprogramming, the short, rod-shaped mitochondria in the embryonic heart gradually transform into large, round mitochondria that gather around myocardial fibers after birth. The morphological maturation of mitochondria in the heart after birth is essential for the improvement of mitochondrial respiratory capacity and the ability to utilize FA, supplying energy for myocardial contraction in the adult heart^1,5^. Furthermore, postnatal cardiac mitochondrial maturation and metabolic reprogramming link together, and any defects in these processes can lead to heart developmental anomalies and even heart failure^5–7^. Another extraordinary change in the postnatal heart is postnatal development. After birth, mouse cardiomyocytes quickly exit from the cell cycle, become bi-nucleated, and undergo hypertrophic growth^8^. Thus, the mouse heart loses regeneration ability within 1 week after birth^9^. However, the mechanisms underlying these changes are largely unknown.

By comparing the gene expression profiles and proteome data of prenatal and postnatal mouse hearts, we found that significant changes in the expression of ketone body metabolism-related genes occurred during the perinatal stage. Both the mRNA and protein levels of *Hmgcs2*, a rate-limiting enzyme in ketogenesis, were increased more than 10 fold in the hearts at postnatal day 7 (P7d) compared with those at embryonic day 18.5 (E18.5d). HMGCS2 localizes in mitochondria and catalyzes the critical step of ketogenesis in adult liver when extrahepatic tissues face energy demand in various physiological and pathological states, such as starvation, post-exercise, diabetes, and obesity^10^. Through ketogenesis, hepatic mitochondria produce ketone bodies, β-hydroxybutyrate (β-HB), acetoacetate, and acetone, from acetyl-CoA at a rate proportional to FA beta-oxidation. β-HB and acetoacetate can be transported into the circulatory system and oxidized by extrahepatic tissues such as the brain and heart, providing energy for their function^10^. Ketogenesis is also evoked by an extremely high-fat diet called ketogenic diet which consists of about 90% fat and 10% protein without carbohydrate^10,11^. Immediately after birth, most mammalian newborns are fed with colostrum, which is also a high-fat, low-carbohydrate diet^12^.

The above analysis inspired us to explore the relationship among ketogenesis, metabolic transition and the loss of heart regeneration capacity during the neonatal stage. We hypothesized that the high fat in colostrum can stimulate ketogenesis in myocytes, which is critical for mitochondrial maturation, the metabolic transition from anaerobic glycolysis to aerobic FA beta-oxidation in mitochondria, and heart regeneration ability loss.

## Results

### Metabolic reprogramming and cell cycle arrest in the neonatal mouse heart are revealed by multi-omics analysis

**Fig. 1:**
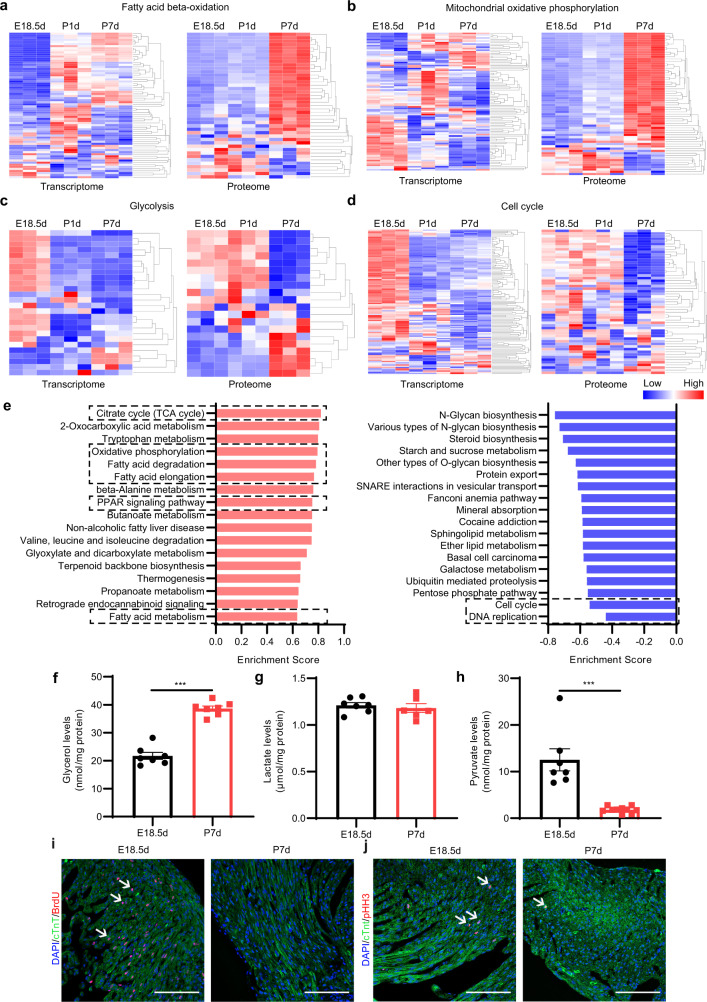
Metabolic reprogramming and cardiomyocyte cell cycle arrest occur in the neonatal mouse heart. **a**–**d** Heatmap of the mRNA and proteins related to FA beta-oxidation (**a**), mitochondrial oxidative phosphorylation (**b**), glycolysis (**c**), and cell cycle (**d**). **e** Gene Set Enrichment Analysis (GSEA) of the proteome in perinatal mouse hearts (P7d vs E18.5d). Left: Positive enrichment score (ES). Right: Negative ES. **f** Glycerol levels in the hearts of E18.5d and P7d mice (*n* = 7 for each group). **g** Lactate levels in the hearts of E18.5d and P7d mice (E18.5d, *n* = 7; P7d, *n* = 6). **h** Pyruvate levels in the hearts of E18.5d and P7d mice (E18.5d, *n* = 7; P7d, *n* = 10). **i** Immunofluorescence staining of cTnT and BrdU in the hearts of E18.5d and P7d mice. Scale bar, 50 μm. The white arrows indicate cTnT and BrdU double-positive cells. **j** Immunofluorescence staining of cTnT and pHH3 in the hearts of E18.5d and P7d mice. Scale bar, 50 μm. The white arrows indicate cTnT and pHH3 double-positive cells. Data are presented as means ± SEM and the *P* values were determined by unpaired two-tailed Student’s *t*-test. ***P < 0.001.

To investigate the genetic and metabolic basis of postnatal heart development, we collected E18.5d, P1d and P7d mouse hearts and performed transcriptomics, proteomics and metabolomics assays. mRNA and protein expression profiles revealed significant changes in metabolic pathways. The expression of most genes involved in FA beta-oxidation and mitochondrial oxidative phosphorylation was largely increased in the neonatal heart (Fig. 1a, b; Supplementary Tables S1 and S2), while the expression of glycolysis- and cell cycle-related genes was significantly decreased (Fig. 1c, d; Supplementary Tables S1 and S2). These expression patterns were further confirmed by real-time quantitative PCR (qPCR) in E18.5d and P7d mouse hearts (Supplementary Fig. S1a–d). KEGG enrichment analysis also showed that the expression of proteins related to FA metabolism, FA degradation and the peroxisome proliferation-activated receptor (PPAR) signaling pathway was largely increased, while the expression of genes involved in cell proliferation, such as cell cycle- and DNA replication-related genes (Fig. 1e), was mostly decreased in neonatal hearts. Gene Ontology (GO) analysis of the proteomics data also suggested that the expression of the mitochondrial component protein (Supplementary Fig. S1e) was largely increased, suggesting the association between mitochondrial maturation and the metabolic pattern transition in the neonatal heart.

Furthermore, the content of glycerol, a triglyceride utilization metabolite, was higher in neonatal hearts than that in fetal hearts (Fig. 1f). However, the level of the glycolysis product, lactate, remained unchanged (Fig. 1g) although the pyruvate level was decreased in neonatal hearts (Fig. 1h), which might be due to an increase in pyruvate metabolism-related gene expression (Fig. 1c; Supplementary Tables S1, S2). A metabolomics study of perinatal hearts indicated that the changes of the metabolites in glucose metabolism were diverse, such as glucose-6-phosphate, phosphoenolpyruvate and lactate (Supplementary Fig. S1f), while the tricarboxylic acid (TCA) cycle remained active since NAD^+^ and NADH levels were augmented during the neonatal stage (Supplementary Fig. S1f and Table S3). On the other hand, as mentioned before, the mRNA levels of most cell cycle marker genes, such as most cyclins and cyclin-dependent kinases were down-regulated (Supplementary Fig. S1d). Moreover, the proportions of BrdU- and phosphorylated histone H3 (pHH3)-positive cardiomyocytes were decreased significantly after birth (Fig. 1i, j; Supplementary Fig. S1g, h), which indicates that the cell cycle of cardiomyocytes is rapidly arrested in the postnatal heart.

The above multiomics analysis suggests the existence of an association among metabolic reprogramming, mitochondrial maturation and cell cycle arrest in the neonatal heart that is critical for the functional transition from the embryonic stage to the adult stage.

### Vigorous ketogenesis occurs in neonatal mouse heart

**Fig. 2:**
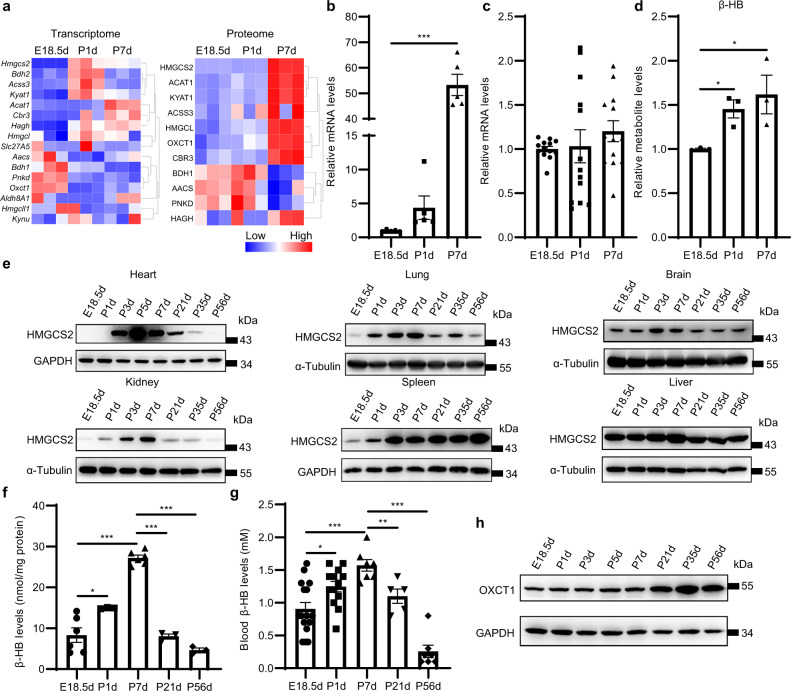
Ketogenesis is enhanced in neonatal mouse heart. **a** Heatmap of the transcription levels (left) and protein levels (right) of genes related to ketone body metabolism in E18.5d, P1d and P7d mouse hearts according to the transcriptomics, proteomics and metabolomics data. **b** qPCR detection of *Hmgcs2* mRNA levels in the hearts of E18.5d, P1d and P7d mice (*n* = 5 for each group). **c** qPCR detection of *Oxct1* mRNA levels in the hearts of E18.5d, P1d and P7d mice (*n* = 13 for each group). **d** Relative β-HB levels in E18.5d, P1d and P7d mouse hearts according to the metabolomics data (*n* = 3 for each group). **e** Western blot detection of the protein levels of HMGCS2 in multiple tissues of mice from the embryonic period to adulthood. **f** β-HB levels in mouse hearts from the embryonic period to adulthood (*n* = 3–6 for each group). **g** Blood β-HB levels of mice from the embryonic period to adulthood (*n* = 5–16 for each group). **h** Western blot detection of the protein levels of OXCT1 in mouse hearts from the embryonic period to adulthood. Data are presented as means ± SEM. and the *P* values were determined by one-way ANOVA. *P < 0.05; **P < 0.01; ***P < 0.001.

In our omics data, we noticed that another group of metabolic genes, ketone body metabolic genes, displayed a striking expression pattern in the neonatal heart. We found that the mRNA and protein levels of *Hmgcs2*, the gene encoding the rate-limiting enzyme in ketogenesis^13^, were increased dramatically in the hearts of neonatal mice compared with those of fetal mice (Fig. 2a, b; Supplementary Fig. S2a, b). Furthermore, though the mRNA level of *Oxct1*, a critical gene responsible for ketone body oxidation, detected by RNA-seq decreased from embryonic to neonatal period, qPCR detection showed that *Oxct1* mRNA did not change (Fig. 2a, c), probably because *Oxct1* changed less and was susceptible to individual differences or due to the quantification error of RNA-seq caused by the rare wrong alignment between the reads and the reference transcriptome^14^. However, both proteomics and western blot revealed that the protein levels of OXCT1 in P7d hearts were higher than those in E18.5d hearts (Fig. 2a; Supplementary Fig. S2a, c). Considering that the increase in HMGCS2 protein expression is much higher than that of OXCT1 from the embryonic to neonatal period, we speculate that ketone bodies accumulate in the neonatal heart. As expected, the metabolomic data indicated that the levels of β-HB in P1d and P7d heart tissues were higher compared with those in E18.5d hearts (Fig. 2d). Examination of *Hmgcs2* mRNA and protein from the fetal stage to the adult stage showed a typical transient expression pattern during the suckling stage. Not only in heart, but also in most other tissues, including the lung, brain, kidney, the expression of HMGCS2 was extremely low in the embryonic stage and rose sharply after birth, reaching a peak from P3d to P7d. Then, it gradually decreased and disappeared after weaning (P21d) in these organs except the liver and spleen (Fig. 2e; Supplementary Fig. S2d, e).

Moreover, HMGCS2 was not detected in either the atria or ventricles of embryonic hearts and was massively expressed in the ventricular wall but not in the atria of P3d and P7d mouse hearts (Supplementary Fig. S2f). Moreover, HMGCS2 was preferred to express in right ventricle and epicardium but it was rarely expressed in the left ventricle and interventricular septum. The contents of β-HB in heart tissues were increased after birth, reached a maximum level at P7d and then decreased after weaning (P21d) (Fig. 2f). Interestingly, the ketone body level in the blood was above 1 mM during all suckling stages, comparable to the levels under some extreme physiological conditions such as prolonged exercise and overnight fasting^10^, and decreased to the normal physiological level by P56d (Fig. 2g). We also found that the mRNA and protein levels of *Oxct1* were dramatically increased after suckling (Fig. 2h; Supplementary Fig. S2g, h), which suggests that the heart can use ketone bodies as energy substrates after suckling.

### Colostrum FA induces transient massive expression of *Hmgcs2* in neonatal mouse heart

**Fig. 3:**
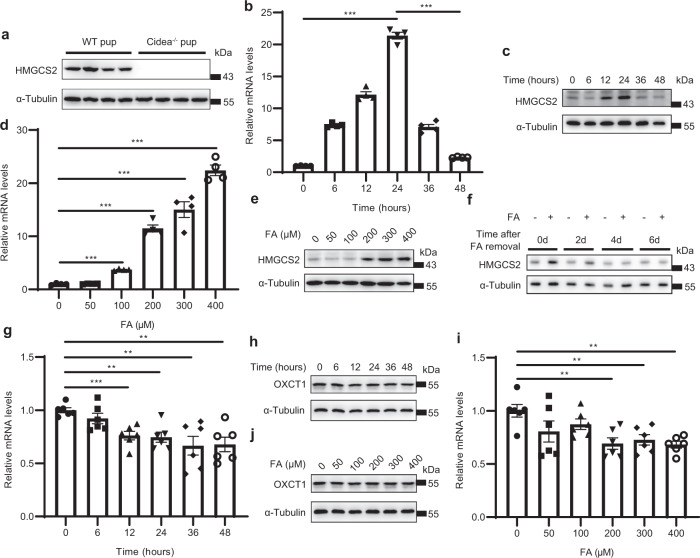
FA in colostrum stimulates transiently abundant *Hmgcs2* expression in neonatal mouse heart. **a** Western blot was performed to detect the expression levels of HMGCS2 in hearts from WT and *Cidea*^−/−^ mouse offsprings (P3d). **b** *Hmgcs2* mRNA levels of NRVMs treated with FA (200 μM) for different times (*n* = 4 for each group). **c** Protein levels of HMGCS2 in NRVMs treated with FA (200 μM) for different times. **d** *Hmgcs2* mRNA levels in NRVMs treated with different doses of FA for 24 h (*n* = 4 for each group). **e** Protein levels of HMGCS2 in NRVMs treated with different doses of FA for 24 h. **f** NRVMs were treated with FA (200 μM) for 24 h, and then the FA in the medium was removed. Then, NRVMs were cultured for 2 days (2d), 4 days (4d), and 6 days (6d), and western blotting was performed to detect HMGCS2 protein levels. **g** *Oxct1* mRNA levels of NRVMs treated with FA (200 μM) for different times (*n* = 6 for each group). **h** Protein levels of OXCT1 in NRVMs treated with FA (200 μM) for different times. **i** *Oxct1* mRNA levels in NRVMs treated with different doses of FA for 24 h (*n* = 6 for each group). **j** Protein levels of OXCT1 in NRVMs treated with different doses of FA for 24 h. Data are presented as means ± SEM. and the *P* values were determined by one-way ANOVA. **P < 0.01; ***P < 0.001.

Then, we explored which factor is responsible for *Hmgcs2* induction during the neonatal stage. There are two significant environmental alterations during perinatal period: the increase in oxygen concentration in the blood because of spontaneous respiration and the introduction of dietary lipids through colostrum^12,15^. Ketone bodies are normally produced at a rate proportional to FA oxidation^10^. We found that the expression of *Hmgcs2* was detectable during all suckling periods and disappeared soon after weaning at approximately the 3^rd^ week after birth. Thus, we hypothesized that colostrum promotes the rapid expression of *Hmgcs2* in the neonatal heart. Cidea is a lipid droplet-associated protein and is abundantly expressed in lactating mammary glands. The deficiency of Cidea reduces milk lipid secretion^16^. We detected the protein levels of HMGCS2 in pups from *Cidea*^−/−^ females and wild-type males and found that there was no HMGCS2 in the heart tissue of the pups of *Cidea*^−/−^ females (Fig. 3a; Supplementary Fig. S3a). Thus, the HMGCS2 expression in the postnatal heart was not able to be induced by lipid-deficient maternal milk. We isolated neonatal rat ventricular myocytes (NRVMs) from the hearts of P0.5d rats that did not suckle milk. Following FA (palmitate (PA): oleate (OA) = 1:1) treatment, we found that both the mRNA and protein levels of *Hmgcs2* in NRVMs were markedly enhanced by FA in a time- (Fig. 3b, c; Supplementary Fig. S3b) and dose- (Fig. 3d, e; Supplementary Fig. S3c) dependent manner. When we withdrew FA from the culture medium, the protein level of HMGCS2 gradually decreased beginning on the 4^th^ day, and almost no HMGCS2 was found after the removal of FA for 6 days (Fig. 3f; Supplementary Fig. S3d). Moreover, FA did not induce OXCT1 expression (Fig. 3g–j; Supplementary Fig. S3e, f), which indicates that FA in colostrum is critical for the transient expression of HMGCS2 but not OXCT1. We also examined the relationship between hypoxia and *Hmgcs2* expression by treating NRVMs with CoCl_2_ to imitate hypoxic conditions. CoCl_2_ did not affect HMGCS2 expression (Supplementary Fig. S3g, h). All the data indicate that the abundant FA in colostrum is responsible for the induction of *Hmgcs2* expression during the suckling period.

### *Hmgcs2* and ketone body β-HB are essential for mitochondrial maturation in neonatal mouse heart

**Fig. 4:**
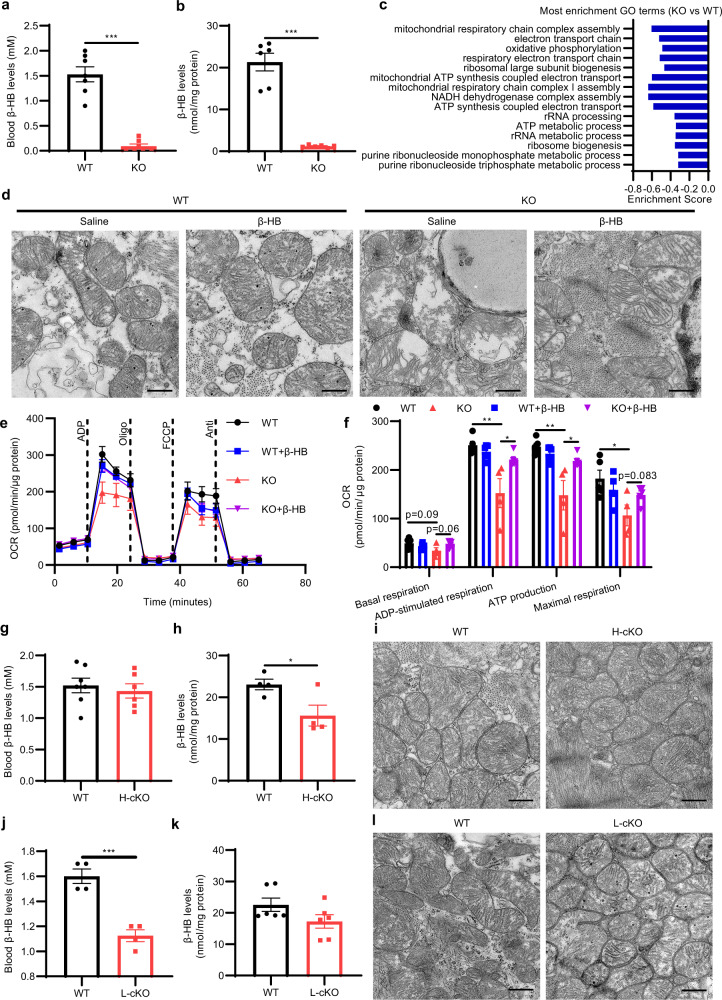
Deletion of ketone bodies disrupts mitochondrial maturation in neonatal mouse heart. **a** Levels of β-HB in the blood of P5d WT and KO mice (*n* = 7 for each group). **b** Levels of β-HB in the hearts of P5d WT and KO mice (*n* = 6 for each group). **c** GSEA was performed to determine the biological processes that were significantly affected, and the ES was analyzed (P3d KO mouse hearts vs WT mouse hearts). **d** The ultrastructures of mitochondria in the hearts of P5d WT and KO mice were observed by transmission electron microscopy. Scale bar, 500 nm. The white star indicates the lipid droplets. **e** The oxygen consumption capacity of isolated mitochondria in the hearts of P5d WT and KO mice treated with saline or β-HB (400 mg/kg/day through intraperitoneal injection (i.p.) from P3d to P5d) was detected by an extracellular metabolic flux analyzer (*n* = 4–5 for each group). **f** Statistical analysis of the OCR in Fig. 4e (*n* = 4–5 for each group). **g** Levels of β-HB in the blood of P5d WT and H-cKO mice (WT, *n* = 7; H-cKO, *n* = 6). **h** Levels of β-HB in the hearts of P5d WT and H-cKO mice (*n* = 4 for each group). **i** The ultrastructures of mitochondria in the hearts of P5d WT and H-cKO mice were observed by transmission electron microscopy. Scale bar, 500 nm. **j** Levels of β-HB in the blood of P5d WT and L-cKO mice (*n* = 4 for each group). **k** Levels of β-HB in the hearts of P5d WT and L-cKO mice (*n* = 6 for each group). **l** The ultrastructures of mitochondria in the hearts of P5d WT and L-cKO mice were observed by transmission electron microscopy. Scale bar, 500 nm. Data are presented as means ± SEM. and the *P* values were determined by unpaired two-tailed Student’s *t*-test. *P < 0.05; **P < 0.01; ***P < 0.001.

To determine the effect of massive *Hmgcs2* expression and ketone body on the status of cardiomyocytes in neonatal hearts, we inactivated *Hmgcs2* gene in all tissues by ubiquitously expressing *EIIA-*Cre (Supplementary Fig. S4a, b). The levels of β-HB in both blood (Fig. 4a) and heart tissues (Fig. 4b) were decreased dramatically. Then we assessed the gene expression profiles in the hearts of neonatal wild-type (WT) and *Hmgcs2* knockout (KO) mice. GSEA indicated that *Hmgcs2* deletion blocked the increased expression of genes in neonatal hearts, such as mitochondrial oxidative phosphorylation- and electron transport chain-related genes (Fig. 4c; Supplementary Fig S4c, d). Meanwhile, the morphology of mitochondria was dramatically altered with disorganized cristae (Fig. 4d) and an increase in the mitochondrial area (Supplementary Fig. S4e). β-HB can significantly alleviate the abnormal mitochondrial morphology caused by *Hmgcs2* knockout, although mitochondrial swelling was not rescued by β-HB (Fig. 4d; Supplementary Fig. S4e). Moreover, lipid droplets were observed in KO mouse hearts (Fig. 4d, star). BODIPY staining also revealed lipid droplet accumulation in KO mouse hearts, which could be alleviated by β-HB (Supplementary Fig. S4f, g). Further functional examination of isolated mitochondria indicated that *Hmgcs2* knockout considerably reduced the mitochondrial oxygen consumption rate (OCR) of neonatal mouse hearts, and β-HB could significantly improve the mitochondrial function inhibited by *Hmgcs2* knockout (Fig. 4e, f). Meanwhile, *Hmgcs2* knockout did not affect mitochondrial DNA copy number (Supplementary Fig. S4h). Thus, *Hmgcs2* and ketone body β-HB are essential for mitochondrial maturation in neonatal mouse hearts.

To further determine whether ketone bodies produced in the heart regulate mitochondrial maturation, we examined the effect of cardiac-specific knockout of *Hmgcs2* on mitochondrial maturation in the heart. Thus, we constructed the heart-specific conditional *Hmgcs2* knockout (H-cKO) mice. The mRNA and protein levels of *Hmgcs2* in the heart were significantly reduced in H-cKO mice (Supplementary Fig. S5a, b). Cardiomyocyte-specific *Hmgcs2* knockout had no significant effect on ketone body levels in the blood (Fig. 4g), possibly because ketogenesis takes place mainly in the liver. The ketone body contents were decreased by approximately 30% in the heart tissues of cardiomyocyte-specific *Hmgcs2* knockout mice compared with those of WT mice (Fig. 4h). Surprisingly, we found that the morphology of mitochondria in the heart was not altered (Fig. 4i), although a decreased mitochondrial area was observed (Supplementary Fig. S5c).

The liver is the main organ producing ketone bodies in the adult body, which can be absorbed and utilized by the heart^10^. Therefore, cardiomyocytes could uptake liver-derived ketone bodies from blood although *Hmgcs2* was deleted in the heart, which resulted in a large amount of residual ketone bodies in the hearts of myocardial-specific *Hmgcs2* knockout mice (Fig. 4h). Thus, this limited ketone body decrease might not cause the change of mitochondrial morphology. To confirm hepatic contribution to ketone body levels in postnatal mouse hearts, we further obtained liver-specific conditional *Hmgcs2* knockout (L-cKO) mice. The mRNA and protein levels of *Hmgcs2* in the liver were significantly reduced in L-cKO mice (Supplementary Fig. S5d, e). It was found that liver-specific knockout of *Hmgcs2* resulted in a decrease in ketone body levels in the blood (Fig. 4j), but did not affect ketone body levels in the heart tissue (Fig. 4k). Thus, there was no significant change in mitochondrial morphology and size in the hearts of L-cKO mice, compared with those of WT mice (Fig. 4l; Supplementary Fig. S5f). Thus, liver-specific deletion of *Hmgcs2* does not influence ketone bodies and mitochondrial maturation in neonatal mouse hearts.

Taken together, the effects of *Hmgcs2* deficiency on mitochondrial morphology and function indicate that *Hmgcs2* and ketone body β-HB is essential for mitochondrial maturation in neonatal mouse hearts.

### *Hmgcs2* and ketone body β-HB regulate FA-stimulated metabolic reprogramming in neonatal mouse heart

**Fig. 5:**
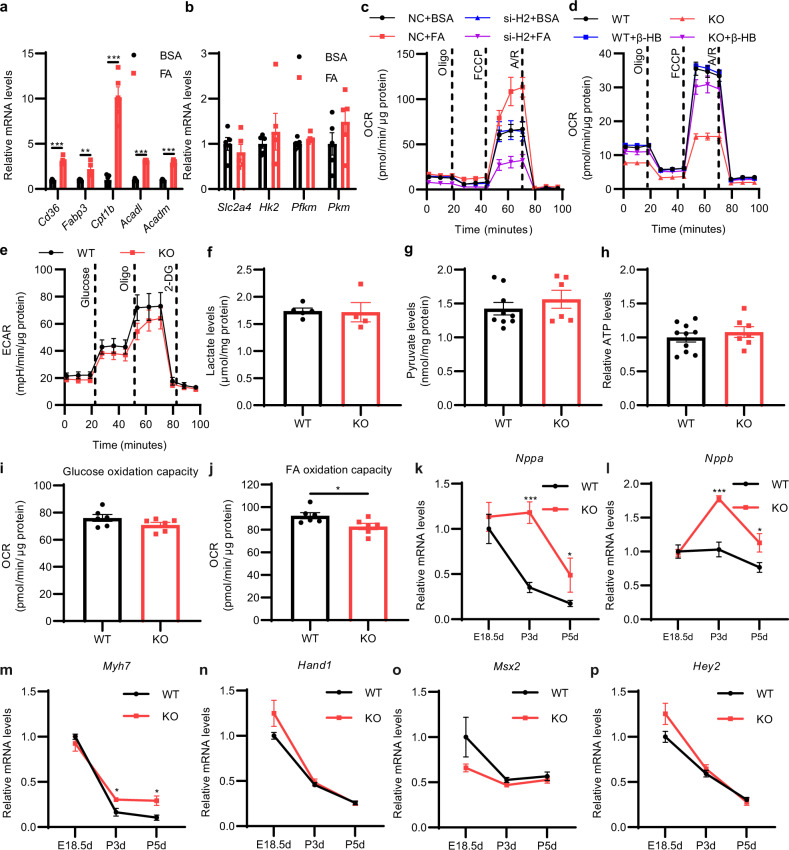
*Hmgcs2* and ketone body β-HB are essential for FA-stimulated metabolic reprogramming in neonatal mouse heart. **a** qPCR detection of the mRNA levels of FA metabolism-related genes in NRVMs treated with BSA or FA (*n* = 5 for each group). **b** qPCR detection of the mRNA levels of glycolytic metabolism-related genes in NRVMs treated with BSA or 200 μM FA (*n* = 5 for each group). **c** Extracellular metabolic flux analysis of the mitochondrial OCR in negative control (NC) and *Hmgcs2*-silenced (si-H2) NRVMs treated with BSA or 200 μM FA (*n* = 4–5 for each group). **d** Extracellular metabolic flux analysis of the mitochondrial OCR in WT and KO neonatal mouse ventricular myocytes (NMVMs) treated with PBS or 5 mM β-HB for 24 h (*n* = 5 for each group). **e** Extracellular metabolic flux analysis of extracellular acidification rate (ECAR) in WT and KO NMVMs (*n* = 5 for each group). **f** Lactate levels in the hearts of P5d WT and KO mice (*n* = 4–5 for each group). **g** Pyruvate levels in the hearts of P5d WT and KO mice (WT, *n* = 9; KO, *n* = 6). **h** Relative ATP levels in the hearts of P5d WT and KO mice (WT, *n* = 10; KO, *n* = 7). **i** Glucose oxidation capacity of the cardiomyocytes in WT and KO mice at P5d was detected by an extracellular metabolic flux analyzer (*n* = 6 for each group). **j** FA oxidation capacity of the cardiomyocytes in WT and KO mice at P5d was detected by an extracellular metabolic flux analyzer (*n* = 6 for each group). **k**–**p** qPCR detection of embryonic marker gene expression in the hearts of WT and KO mice. Data are presented as means ± SEM. and the *P* values were determined by unpaired two-tailed Student’s *t*-test. *P < 0.05; **P < 0.01; ***P < 0.001.

The energy for the fetal heart is supplied mainly by carbohydrates (especially lactate and glucose) from the placenta. Following birth, exposure to lipid-rich colostrum makes it possible for cardiomyocytes to utilize more efficient FA to produce ATP through FA oxidation and oxidative phosphorylation in mitochondria^2^. We found that FA stimulation largely enhanced lipid metabolism in the neonatal heart, since the expression of genes responsible for lipid uptake into cells (*Cd36*, *Fabp3*), lipid uptake into mitochondria (*Cpt1b*) and beta-oxidation (*Acadl and Acadm*) was significantly augmented (Fig. 5a), while the expression of genes responsible for glucose uptake (*Slc2a4*) and metabolism (*Hk2*, *Pfkm*, *Pkm*) was not altered (Fig. 5b). Extracellular metabolic flux analysis indicated that FA stimulation largely enhanced the OCR of cardiomyocytes, but this effect was blocked by *Hmgcs2* knockdown (Fig. 5c; Supplementary Fig. S6a–d). FA-enhanced ATP production was also decreased by *Hmgcs2* knockdown (Supplementary Fig. S6e). *Hmgcs2* deletion also largely decreased the OCR of cardiomyocytes in the neonatal heart, which could be rescued by β-HB (Fig. 5d; Supplementary Fig. S6f). On the other hand, *Hmgcs2* deletion had no significant effect on glycolysis (Fig. 5e; Supplementary Fig. S6g). The lactate, pyruvate and ATP levels were also similar between WT and KO hearts (Fig. 5f–h).

To further clarify the effect of *Hmgcs2* on the ability of cardiomyocytes to utilize metabolic substrates (glucose and FA), we isolated cardiomyocytes from neonatal WT and KO mice and examined metabolic capacity utilizing FA or glucose. Extracellular flux analysis was performed to determine the fuel capacity of FA or glucose by detecting the OCR of a specific fuel when other fuel pathways are inhibited. It was found that *Hmgcs2* knockout had no effect on glucose oxidation capacity but resulted in reduced FA oxidation capacity in cardiomyocytes (Fig. 5i, j). These results are consistent with the previous data that *Hmgcs2* knockout caused lipid accumulation but did not affect pyruvate and lactate levels in mouse hearts (Fig. 5f, g; Supplementary Fig. S4f, g). Therefore, *Hmgcs2* deletion disrupts lipid metabolism and mitochondrial maturation without influencing glucose metabolism in the postnatal heart.

To determine whether the physiological state of the heart of KO mice is close to that of the embryonic heart, we examined the expression of the embryonic marker genes that are strongly expressed in the embryonic heart and are responsible for embryonic heart development. We found that *Hmgcs2* deletion led to the increase of the expression of *Nppa, Nppb*, and *Myh7* rather than *Hand1*, *Msx2* and *Hey2* (Fig. 5k–p). Considering that *Nppa*, *Nppb* and *Myh7* are not only embryonic marker genes but also markers of cardiac hypertrophy, we infer that *Hmgcs2* knockout causes cardiac stress, but does not affect cardiac development. Taken together, all these data suggest that elevations in the expression of *Hmgcs2* and its product β-HB in the neonatal heart are responsible for the metabolic maturation of FA oxidation in the neonatal heart stimulated by FA.

### Neonatal elevation of ketone bodies is critical for postnatal heart development and heart function

**Fig. 6:**
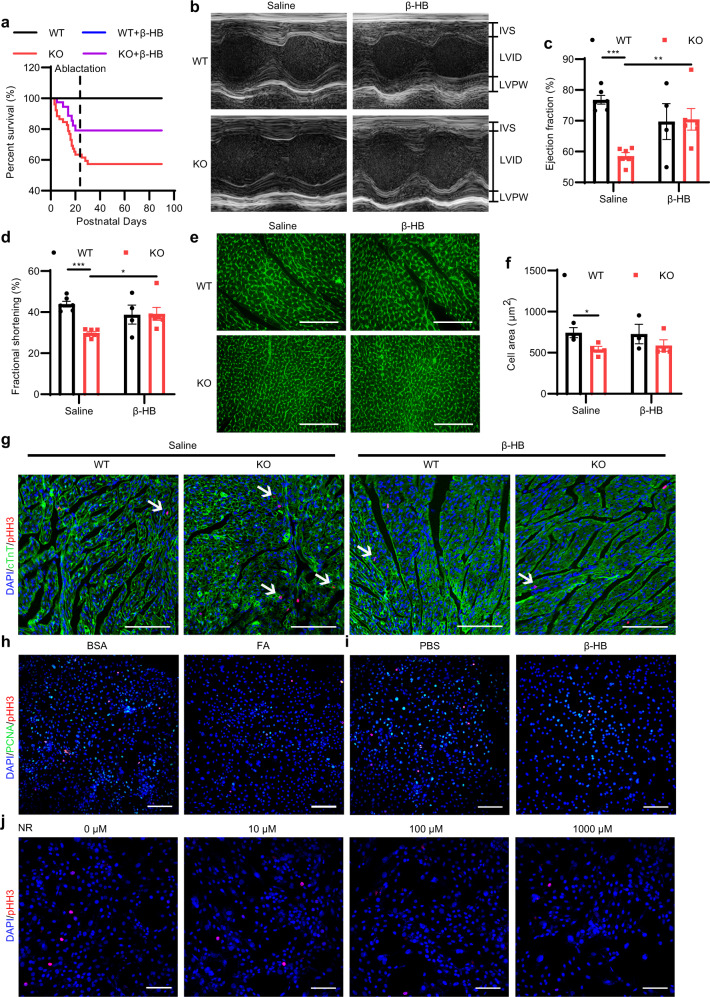
Neonatal elevation of ketone bodies regulates postnatal heart development and heart function. **a**–**e** WT and KO mice were treated with saline or β-HB (400 mg/kg/day) though i.p. from P3d to P21d. **a** Survival rates of WT and KO mice treated with saline or β-HB (WT, *n* = 59; KO, *n* = 52; WT + β-HB, *n* = 43; KO + β-HB, *n* = 42). **b** Visible images of echocardiographic measurements of WT and KO mice treated with saline or β-HB (P21d). **c** Ejection fractions of WT and KO mice treated with saline or β-HB (P21d) (WT + Saline, *n* = 6; KO + Saline, *n* = 6; WT + β-HB, *n* = 4; KO + β-HB, *n* = 6). **d** Fractional shortening of WT and KO mice treated with saline or β-HB (P21d) (WT + Saline, *n* = 6; KO + Saline, *n* = 6; WT + β-HB, *n* = 4; KO + β-HB, *n* = 6). **e** WGA staining of WT and KO mouse hearts treated with saline or β-HB (P21d). Scale bar, 100 µm. **f** Statistics of the cross-sectional area of a single cardiomyocyte in Fig. 6e (WT + Saline, *n* = 3; KO + Saline, *n* = 4; WT + β-HB, *n* = 3; KO + β-HB, *n* = 4). **g** Immunofluorescence staining of pHH3 and cTnT in the hearts of P5d WT and KO mice treated with saline or β-HB (400 mg/kg/day though i.p. from P3d to P5d). The white arrows indicate cTnT and pHH3 double-positive cells. Scale bar, 100 µm. **h** Immunofluorescence staining of pHH3 and PCNA in H9C2 cells treated with BSA or 200 μM FA for 24 h. Scale bar, 200 µm. **i** Immunofluorescence staining of pHH3 and PCNA in H9C2 cells treated with PBS or 5 mM β-HB for 24 h. Scale bar, 200 µm. **j** Immunofluorescence staining of pHH3 in H9C2 cells treated with DMSO or NR for 24 h. Scale bar, 100 µm. Data are presented as means ± SEM. and the *P* values were determined by unpaired two-tailed Student’s *t*-test. *P < 0.05; **P < 0.01; ***P < 0.001.

Next, we explored the consequences of *Hmgcs2* deletion and ketone body depletion during the neonatal stage. We found that only approximately 60% of newborn KO mice survived beyond the suckling period (P21d) (Fig. 6a). When we supplied β-HB during the whole suckling period, the survival percentage increased to 80% (Fig. 6a). Further examination indicated that the heart function of surviving KO mice was largely impaired (Fig. 6b). The ejection fraction (Fig. 6c) and fractional shortening (Fig. 6d) of surviving KO mice were significantly lower than those of WT mice. The anatomic study showed that the heart/body weight ratio showed no significant difference between KO and WT mice (Supplementary Fig. S7a–c), and there were no significant structural differences except for a slight decrease in ventricular wall diameter in the KO mouse hearts (Supplementary Fig. S7d). WGA staining revealed that the cardiomyocyte area was significantly reduced in the ventricular wall of KO mouse hearts (Fig. 6e, f). Moreover, the decrease in cardiac function of KO mouse hearts was alleviated by supplementation with β-HB during the whole suckling period (Fig. 6b–d). However, the structural defects and decreased cardiomyocyte area of KO mouse hearts were not alleviated by β-HB supplementation (Fig. 6e, f; Supplementary Fig. S7d). We also tested the effect of *Hmgcs2* in the liver on the heart function to figure out whether the liver-derived ketone body regulates postnatal heart function. It was found that liver-specific *Hmgcs2* knockout does not influence the heart function of postnatal mice (Supplementary Fig. S7e–g). All these data suggest that a shortage of ketone body stimulation during the neonatal stage results in postnatal heart functional defects.

On the other hand, one of the important events in postnatal heart development is the gradual loss of regeneration ability of the neonatal heart, which occurs within one week after birth^9^. By pHH3 immunofluorescence staining and BrdU incorporation assay, we showed that cardiomyocyte proliferation did not stop in the neonatal hearts of *Hmgcs2*-deficient mice, but it did stop upon administration of β-HB (Fig. 6g; Supplementary Fig. S7h–j). We found that FA in colostrum stimulated the expression of *Hmgcs2* and enhanced mitochondrial function in NRVMs (Figs. 3, 5). Thus, through CCK-8 detection and immunofluorescence staining of pHH3 and proliferating cell nuclear antigen (PCNA), we examined the effect of FA on cardiomyocyte proliferation and found that FA blocked the proliferation of H9C2 cells (Fig. 6h; Supplementary Fig. S7k–m). Moreover, β-HB had a similar effect as FA on cardiomyocyte proliferation (Fig. 6i; Supplementary Fig. S7n–p).

Furthermore, we used nicotinamide riboside (NR) and omaveloxolone (OMA) to enhance mitochondrial activity (Supplementary Fig. S8a, b). Immunofluorescence staining of pHH3 and PCNA showed that cardiomyocyte proliferation was blocked by NR and OMA (Fig. 6j; Supplementary Fig. S8c–i), suggesting that FA-regulated mitochondrial maturation was also related to the inhibition of cardiomyocyte proliferation. Therefore, our data suggest that *Hmgcs2* expression and ketone body production in the neonatal heart are beneficial for postnatal heart development and function.

### *Hmgcs2* and ketone body β-HB regulate mitochondrial maturation possibly by mediating protein β-hydroxybutyrylation and acetylation in the neonatal heart

**Fig. 7:**
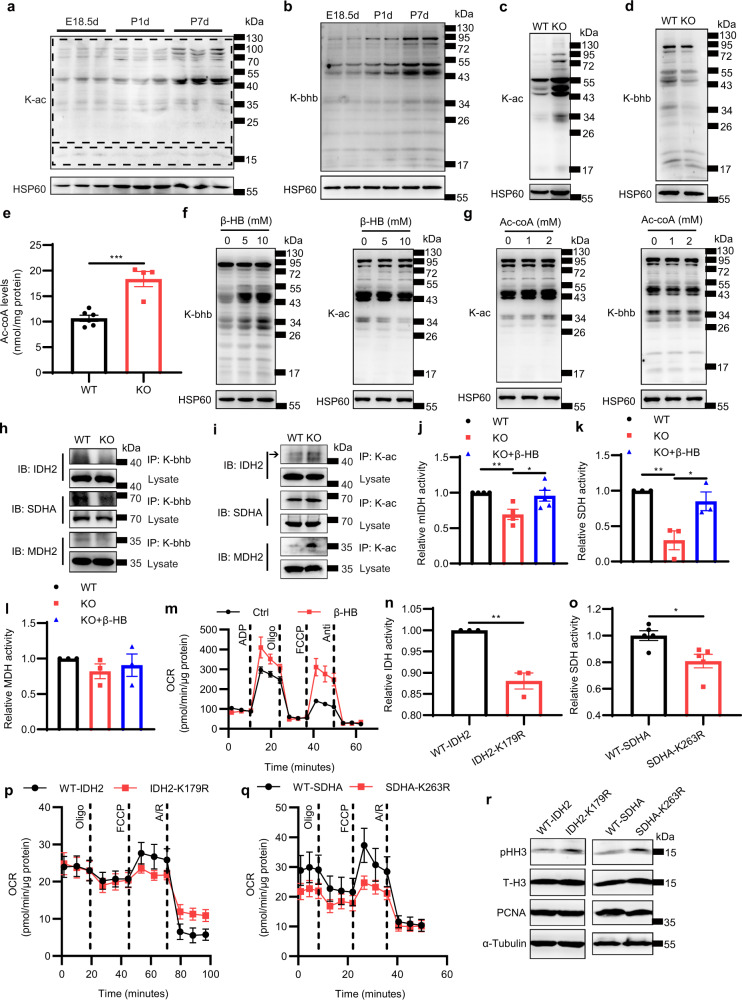
Mitochondrial protein β-hydroxybutyrylation and acetylation may be responsible for the regulation of mitochondrial maturation by ketone body β-HB. **a** Western blot detection of mitochondrial protein K-ac in the hearts of WT mice at E18.5d, P1d and P7d. **b** Western blot detection of mitochondrial protein K-bhb in the hearts of WT mice at E18.5d, P1d and P7d. **c** Western blot detection of mitochondrial protein K-ac in the hearts of WT and KO mice at P5d. **d** Western blot detection of mitochondrial protein K-bhb in the hearts of WT and KO mice at P5d. **e** Levels of Ac-CoA in the hearts of WT and KO mice at P5d (WT, *n* = 6; KO, *n* = 4). **f** Western blot detection of protein K-ac and K-bhb of mitochondria isolated from KO hearts at P5d treated with ddH_2_O or β-HB. **g** Western blot detection of protein K-ac and K-bhb of mitochondria isolated from WT hearts at P5d treated with ddH_2_O or Ac-CoA. **h** Immunoprecipitation assays on WT and KO hearts at P5d. IP: Anti-K-hbb. IB: IDH2, SDHA, MDH2. **i** Immunoprecipitation assays on WT and KO hearts at P5d. IP: Anti-K-ac. IB: IDH2, SDHA, MDH2. **j** Relative mitochondrial IDH (mIDH) activity of mitochondria isolated from WT and KO hearts at P5d treated with ddH_2_O or 5 mM β-HB (WT, *n* = 4; KO, *n* = 4; KO + β-HB, *n* = 5). **k** Relative SDH activity of mitochondria isolated from WT and KO hearts at P5d treated with ddH_2_O or 5 mM β-HB (*n* = 3 for each group). **l** Relative MDH activity of mitochondria isolated from WT and KO hearts at P5d treated with ddH_2_O or 5 mM β-HB (*n* = 3 for each group). **m** The OCR of isolated mitochondria in KO mouse hearts at P5d treated with ddH_2_O or 5 mM β-HB was detected by an extracellular metabolic flux analyzer (*n* = 3 for each group). **n** Relative mIDH activity of mitochondria isolated from 293T cell lines transfected with WT-IDH2 or IDH2-K179R (*n* = 3 for each group). **o** Relative SDH activity of 293T cell lines transfected with WT-SDHA or SDHA-K263R (*n* = 5 for each group). **p** The OCR of 293T cell lines transfected with WT-IDH2 or IDH2-K179R (*n* = 6 for each group). **q** The OCR of 293T cell lines transfected with WT-SDHA or SDHA-K263R (WT-SDHA, *n* = 5; SDHA-K263R, *n* = 6). **r** Western blot detection of pHH3, total histone H3 (T-H3) and PCNA in 293T cell lines transfected with WT-IDH2, IDH2-K179R, WT-SDHA or SDHA-K263R. Data are presented as means ± SEM. and the *P* values were determined by unpaired two-tailed Student’s *t*-test. *P < 0.05; **P < 0.01; ***P < 0.001.

Previous reports have shown that ketogenesis can affect mitochondrial protein lysine acetylation (K-ac) and β-hydroxybutyrylation (K-bhb)^17,18^. Therefore, we isolated mitochondria from perinatal mouse hearts and found that the K-ac of the large-molecular-weight mitochondrial proteins was increased in neonatal hearts compared with that in embryonic hearts. However, the K-ac of the small-molecular-weight mitochondrial proteins did not change (Fig. 7a; Supplementary Fig. S9a). Moreover, the K-bhb of mitochondrial proteins was largely enhanced in the mouse hearts from the embryonic to neonatal period (Fig. 7b; Supplementary Fig. S9b). We then deleted *Hmgcs2* to deplete ketone bodies and found that mitochondrial protein K-ac was increased (Fig. 7c; Supplementary Fig. S9c), while mitochondrial protein K-bhb was largely decreased (Fig. 7d; Supplementary Fig. S9d) in *Hmgcs2*-deficient hearts. Acetyl-CoA and β-HB are donors for protein K-ac and K-bhb, respectively^19,20^. *Hmgcs2* deficiency decreased ketone body levels (Fig. 4b) and led to the accumulation of acetyl-CoA in neonatal hearts (Fig. 7e). Thus, we wondered whether mitochondrial protein K-bhb and K-ac can influence each other. Mitochondria of neonatal mouse hearts were isolated and treated with β-HB or acetyl-CoA. As expected, β-HB promoted mitochondrial protein K-bhb in *Hmgcs2*-deleted hearts (Fig. 7f; Supplementary Fig. S9e). Interestingly, the K-ac of mitochondrial proteins was blocked by β-HB (Fig. 7f; Supplementary Fig. S9f). On the other hand, acetyl-CoA enhanced mitochondrial protein K-ac (Fig. 7g; Supplementary Fig. S9g) but inhibited K-bhb (Fig. 7g; Supplementary Fig. S9h) in WT hearts. The succinate dehydrogenase complex flavoprotein subunit A (SDHA), isocitrate dehydrogenase 2 (IDH2) and malate dehydrogenase 2 (MDH2) are critical enzymes in the TCA cycle and are modified by K-ac and K-bhb^19,21^. In *Hmgcs2*-deficient hearts, the K-bhb of these three enzymes was decreased (Fig. 7h; Supplementary Fig. S9i). However, the K-ac of IDH2, SDHA and MDH2 was up-regulated in KO mouse hearts compared to that in WT mouse hearts (Fig. 7i; Supplementary Fig. S9j). In addition, β-HB could promote K-bhb of IDH2, SDHA rather than MDH2 in KO mouse hearts (Supplementary Fig. S9k). β-HB could also inhibit the increased K-ac of IDH2 and SDHA in KO mouse hearts (Supplementary Fig. S9l). Furthermore, the enzyme activity of IDH and SDH, but not MDH, was decreased in *Hmgcs2*-deleted hearts and recovered by β-HB supplementation (Fig. 7j–l). The mitochondrial respiratory capacity in *Hmgcs2*-deleted hearts was also improved by β-HB (Fig. 7m; Supplementary Fig. S10a).

To further determine the contribution of K-bhb modification induced by β-HB to the activities of IDH, SDH and MDH, the activities of mitochondrial IDH, SDH and MDH in β-HB-treated 293T cells were detected. It was found that the activities of IDH and SDH but not MDH were enhanced by β-HB treatment (Supplementary Fig. S10b–d). Moreover, we mutated the specific lysine residue modified by K-bhb in IDH2 and SDHA^22^. We found that mutations of lysine179 (K179) to arginine (K179R) in IDH2 and lysine263 (K263) to arginine (K263R) in SDHA, which prevented K-bhb, inhibited the enzyme activity of IDH and SDH (Fig. 7n, o). The K to R mutations in IDH2 and SDHA also decreased mitochondrial respiratory capacity (Fig. 7p, q; Supplementary Fig. S10e, f). However, since we cannot rule out the effects of other protein modifications or allosteric effects on enzyme activity, we conclude that ketone body β-HB can promote mitochondrial metabolic enzyme activity and mitochondrial respiratory function, possibly by not only affecting the K-bhb and K-ac modification of mitochondrial proteins, but also other protein modifications and allosteric effect of β-HB. Regarding cell proliferation, PCNA expression was not affected by K to R mutation. However, the protein level of pHH3 was increased by K to R mutation even though the difference was not statistically significant (*P* = 0.062 between WT-IDH2 and IDH2-K179R, *P* = 0.084 between WT-SDHA and SDHA-K263R, respectively. Figure 7r; Supplementary Fig. S10g–h). To exclude the effect of HMGCS2 or ketone body β-HB on the mRNA and protein levels of IDH2, SDHA and MDH2, we detected the mRNA and protein levels of IDH2, SDHA and MDH2, and found that there was no significant difference in the mRNA and protein levels of IDH2, SDHA and MDH2 between WT and KO mouse hearts (Supplementary Fig. S10i, j). Taken together, the results indicate that ketone body β-HB regulates mitochondrial enzyme activity, which might be dependent on mitochondrial protein K-bhb and K-ac (Supplementary Fig. S11).

## Discussion

Organ development continues after birth. For example, the nervous system undergoes myelination and synaptogenesis during the postnatal stage^23^, and the heart also starts hypertrophic growth to prepare for increasing contraction workload during adulthood^24,25^. It has been reported that exposure to famine in infants leads to increased susceptibility to coronary heart disease, hypertension or diabetes in adulthood^26–28^. However, the underlying mechanism in postnatal development remains largely unknown. Herein, our study indicated that *Hmgcs2*-controlled ketone body β-HB might be the important molecule that regulates postnatal heart development by promoting cardiomyocyte mitochondrial maturation and metabolic reprogramming.

External environmental factors such as temperature and oxygen change dramatically during the perinatal stage, and factors related to the internal milieu, such as metabolic substrates and related endocrine hormones, are largely altered after birth. FA levels in the blood are increased immediately after birth due to the consumption of lipid-rich colostrum^29^. FA activates PPAR signaling, permitting the transcription of genes related to FA metabolism, such as FA uptake- and beta-oxidation-related genes^30,31^ as well as ketogenic genes. Ketogenesis occurs at rates proportional to FA oxidation in hepatic mitochondria^10^. We demonstrated that increased FA levels after birth induced *Hmgcs2* expression in cardiomyocytes, which regulated postnatal heart development and cardiac functional maturation. When *Hmgcs2* was deleted to decrease ketone body levels, approximately 40% of mice died before weaning. Sudden cardiac death (SCD) is a rare clinical encounter, but autopsy-negative SCD without heart structure defects is more common in pediatric patients than in adult patients. Recently, sudden arrhythmic death syndrome (SADS) and sudden unexpected death in epilepsy (SUDEP) have emerged as etiologies of nonstructural heart defect SCD in children and adults^32^. However, we still do not know how SADS and SUDEP developed in these individuals. The early death of *Hmgcs2-*deleted mice and the cardiac function defects of surviving mice suggest that postnatal heart development regulated by ketone body might be related to the etiology of SCD.

We found in Fig. 2 that only HMGCS2 in the liver and spleen was consistently expressed after birth. High abundance of HMGCS2 expression in liver is reasonable since liver is responsible for ketone body production in adult stage^10^. The postnatal spleen continues to develop and gradually improves its immune function^33^. After birth, aerobic respiration provides most of the energy required by the body. In this work, we found that ketone bodies are essential for mitochondrial maturation after birth. Moreover, a previous study found that ketone bodies can promote the development of memory T cells^34^. Thus, the persistent expression of HMGCS2 in the postnatal spleen may be related to the postnatal development of the spleen.

HMGCS2 was highly expressed in the right ventricle and epicardium but it was rarely expressed in the left ventricle and interventricular septum. We infer that there might be some relationship between HMGCS2 expression pattern and heart function. Most of the energy required for healthy adult mammalian heart contraction comes from fatty acids, with the remainder being derived from glycolysis^35^. Although no study has found a relationship between heart failure and HMGCS2 expression, a failing heart has a reduced ability to utilize fatty acids and tends to utilize ketone bodies^36^. In addition, compared with the left ventricle, the right ventricle has a weaker systolic function and has a relatively lower demand for fatty acid utilization^37^. Moreover, the epicardium has no systolic function. Therefore, we speculate that the high expression of HMGCS2 in the right ventricle and epicardium may be related to weak heart function.

Although the predominant pathways of energy production in the heart are FA catabolism and glucose/lactate catabolism, cardiomyocytes can also utilize ketone bodies^10^. The exciting finding of ketone metabolism suggests that failing human hearts preferentially switch to ketone body utilization^38–40^. An increasing number of studies have supported the notion that ketone bodies are not only metabolic fuel for cardiac mitochondrial oxidation but also important regulatory molecules that play a role in both signal transduction and epigenetic modification^10,41^. Here, we showed that ketone body deficiency caused by *Hmgcs2* knockout blocked postnatal mitochondrial maturation by directly modifying mitochondrial proteins and downregulating their enzymatic activity. The impairment of mitochondrial function led to a decrease in FA oxidation in cardiomyocytes. Thus, we observed lipid accumulation in *Hmgcs2* knockout hearts. Consistent with our study, a study on human diseases with *HMGCS2* mutations revealed that mutations in *HMGCS2* led to symptoms of fatty liver and hepatomegaly in patients^42^, which might be because that the loss of ketogenic function of HMGCS2 blocked the metabolic flow channeled from acetyl-CoA to ketone bodies and thus inhibited lipid oxidation. A recent study showed that inhibiting FA oxidation can promote cardiomyocyte proliferation after birth^43^. We did find that ketone body inhibited cardiomyocyte proliferation after birth, possibly through regulation of FA metabolism in the neonatal heart. Thus, our observation suggests that FA in colostrum-stimulated ketogenesis is critical for heart functional maturation or postnatal development.

Since the expression of ketone body oxidative genes such as *Oxct1* is not elevated by FA, the ketone bodies whose synthesis is increased by *Hmgcs2* might not be metabolic fuel but important regulatory molecules^20^. Here, we revealed that the modification of mitochondrial proteins was affected by *Hmgcs2* deletion: protein K-bhb was inhibited, while protein K-ac was enhanced by *Hmgcs2* knockout in neonatal hearts. It has been reported that approximately 60% of mitochondrial proteins contain lysine acetylation sites, and K-ac inhibits the enzyme activity of most mitochondrial proteins^19^. Ketogenesis deficiency in the liver leads to hepatic mitochondrial dysfunction and lipid accumulation during the neonatal period due to mitochondrial protein hyperacetylation^44^. Moreover, a recent study indicated that K-bhb can enhance the enzyme activity of citrate synthase^18^. However, this study did not explore the effect of K-bhb on the other enzymes in mitochondria and the relationship between K-bhb and K-ac. Our work indicates that the activity of various mitochondrial enzymes such as IDH and SDH relies on K-bhb. Furthermore, we found that β-HB inhibited mitochondrial protein K-ac, while acetyl-CoA inhibited mitochondrial protein K-bhb. This suggests that K-bhb and K-ac of mitochondrial proteins might compete. The balance of protein K-bhb and K-ac might regulate the functions of mitochondrial proteins, such as IDH and SDH, and then affect mitochondrial maturation. However, even though K to R mutation inactivated protein K-bhb^18^, whether K-bhb and K-ac compete at the same lysine sites of mitochondrial proteins needs more examination in the future.

It has been reported that cardiomyocytes of failed hearts generally have a reduced ability to utilize FA and thus tend to absorb and utilize ketone bodies^36,45^. β-HB intervention could be used to maintain heart function in heart failure patients^46^. Under conditions of ischemia or reperfusion injury, a ketogenic diet or ketone bodies have cardioprotective effects, possibly because ketone bodies treatment increases the abundance of cardiac mitochondria^47–50^. Since elevations in ketone bodies are beneficial to postnatal heart function, our study suggests that supplementation with ketone bodies may alleviate abnormal heart development or function in babies after birth. Furthermore, we speculate that ketone body deficiency during the postnatal stage might be related to the incidence of sudden cardiac death in infants and adolescents^51^. Thus, ketone body supplementation may also be an effective treatment for preventing sudden cardiac death.

In conclusion, we have revealed that transient ketone body elevation in the postnatal heart can enhance K-bhb and inhibit K-ac of mitochondrial proteins, which might be essential for mitochondrial maturation and cardiac function in postnatal mice.

## Materials and methods

### Animals

C57BL/6J mice were obtained from GemPharmatech Co., Ltd. (Jiangsu, China). *Cidea* knockout mice were obtained from Peng Li laboratory in Tsinghua University. CRISPR/Cas9 system was used to make *Hmgcs2*-floxed mice (*Hmgcs2*^*fl/fl*^) mice. In detail, Cas9 mRNA, sgRNA and donor were co-injected into zygotes. sgRNA directed Cas9 endonuclease cleavage in intron 1–2 and intron 2–3, and created a double-strand break. Such breaks were then repaired, and loxp sites were inserted into intron 1–2 and intron 2–3 respectively by homologous recombination. The sequence between two loxp sites will be deleted in specific tissues or cells when tissue-specific promoter drives the expression of Cre. Systemic *Hmgcs2* knockout mice were generated by crossing *Hmgcs2*^*fl/fl*^ mice with *EIIA-*Cre transgenic mice. H-cKO mice were generated by crossing *Hmgcs2*^*fl/fl*^ mice with *cTnT-*Cre transgenic mice. L-cKO mice were generated by crossing *Hmgcs2*^*fl/fl*^ mice with *Alb-*Cre transgenic mice. All animals were housed in an environment with strictly controlled temperature and humidity under a normal 12-h light/dark cycle and were fed ad libitum. All animal experiments were carried out according to the guidelines of the Animal Care and Use Committee of Nanjing Medical University in Nanjing, China.

The hearts of WT and *Hmgcs2* knockout mice at P3d were collected for transcriptomic analysis. C57BL/6J mice at E18.5d, P1d and P7d used for multi-omics analysis were obtained from the Nanjing Medical University animal care unit (Nanjing, China). The mouse hearts were collected for transcriptome, proteome and metabolome analysis. Each time point had three biological replicates.

Neonatal mice were treated with saline or β-HB (400 mg/kg/day) through i.p. from P3d to P5d or from P3d to P21d. 1 hour before the mice were sacrificed, neonatal mice were treated with BrdU (50 mg/kg) through i.p.

### Cell culture

The hearts of neonatal (0.5-day-old) Sprague–Dawley rats were dissociated and digested with trypsin and type II collagenase (Gibco, USA) to obtain NRVMs. The NRVMs were attached to the bottom of the cell culture dish 48 h after plating. NRVMs or H9C2 cells were cultured in Dulbecco’s modified Eagle’s medium (DMEM, Sigma-Aldrich, cat D7777, USA) supplemented with 10% fetal bovine serum (FBS, Gibco, USA), 100 U/mL penicillin and 100 μg/mL streptomycin at 37 °C with 5% CO_2_. The method used to isolate NMVMs was the same as that used to isolate NRVMs.

siRNA was transfected into NRVMs using Lipofectamine 2000 reagent (Invitrogen, cat 11668019, USA) for 48 h according to the manufacturer’s instructions.

FA (palmitate (PA): oleate (OA) = 1:1, 200 μM) was used to treat NRVMs for indicated times, then mRNA and protein levels of HMGCS2 were detected. Both PA and OA were dissolved in 10% BSA.

### Chemicals

Palmitate (cat P5585), oleate (cat O7501), FA-free bovine serum albumin (BSA, cat A0281), CoCl_2_ (cat C8661), β-HB (cat H0261), BrdU (cat B5002) and Ac-coA (cat A2181) were purchased from Sigma-Aldrich (USA). NR (cat S2935) and OMA (cat S7672) were purchased from Selleck (China).

### Measurement of β-HB, lactate, pyruvate, triglycerides, and ATP

The content of β-HB in blood was detected by a blood ketone tester (Keto Mojo, USA). The content of β-HB in heart tissues was detected using a β-Hydroxybutyrate Colorimetric Assay Kit (BioVision, cat K632-100, USA) according to the manufacturer’s instructions. The lactate, pyruvate, and triglyceride levels in heart tissues were measured using a detection kit purchased from Nanjing Jiancheng Bioengineering Institute (cat A019-2, A081, and A110-1, China) according to the manufacturer’s instructions. The ATP content in heart tissues was detected using a detection kit purchased from Beyotime (cat S0026, China) according to the manufacturer’s instructions.

### Echocardiographic measurements

The mice were anesthetized with isoflurane. Then, cardiac function was assessed by transthoracic two-dimensional guided M-mode echocardiography with a Vevo 2100 (VisualSonics, Canada).

### RNA isolation and qPCR

Total RNA was isolated from mouse hearts or cells using TRIzol reagent (Takara, cat 9109, Japan) according to the manufacturer’s instructions. The concentration of total RNA was measured with a NanoDrop 2000 (Thermo Fisher Scientific, USA). Then, PrimeScript™ RT Master Mix (Takara, Japan) was used to reverse-transcribe 1 μg of RNA into cDNA according to the manufacturer’s instructions.

qPCR was performed with ChamQ Universal SYBR qPCR Master Mix (Q711, Vazyme Biotech Co., Ltd, China) on a ViiA 7 Real-Time PCR System (Applied Biosystems, USA). The relative mRNA levels were normalized to those of *Rplp0* (mouse) or *Rn18s* (rat). The primer sequences are listed in Supplementary Table S4.

### Mitochondrial genome quantification

Total DNA of the hearts was extracted using Phenol-Chloroform-Isoamylol (Solarbio, P1012, China). mtDNA content was assessed by qPCR using primers specific for mitochondrial-encoded genes (*mt-Co1, mt-Dloop, mt-Atp6*) and normalized to nuclear DNA content (a specific locus on mouse chromosome 6). Primer sequences are listed in Supplementary Table S4.

### Histological analysis, immunohistochemistry, and immunofluorescence staining

Mouse hearts from different developmental stages were collected and routinely fixed in 4% paraformaldehyde (PFA) overnight, dehydrated in gradient ethanol, embedded in paraffin wax, and sectioned into 5 μm slices.

For histological analysis, the paraffin sections of heart tissues were stained with hematoxylin and eosin (H&E) according to a standard protocol.

For immunohistochemistry and immunofluorescence staining, the paraffin sections were dewaxed, rehydrated, and boiled in citrate buffer (pH 6.0) for antigen retrieval. Then, the sections were permeabilized with 0.1% Triton X-100, blocked with 10% goat serum and incubated with the indicated primary antibodies overnight at 4 °C. Afterward, the slides were washed with PBS 3 times, incubated with secondary antibodies for 1–2 h at room temperature and then washed with PBS 3 times. Immunohistochemical detection was performed with a DAB substrate kit (ZSGB-BIO, cat ZLI-9018, China). A FluoView™ FV3000 confocal microscope (Olympus, Japan) was used to observe the immunofluorescence staining.

The primary antibodies used in immunohistochemistry and immunofluorescence staining are listed in Supplementary Table S5.

### Transmission electron microscopy

Mouse hearts were fixed in 4% glutaraldehyde, postfixed in 1% osmium tetroxide, dehydrated in gradient acetone, and embedded in Epon resin. The resin was then polymerized at 30 °C, 45 °C, and 60 °C for 1 day per temperature. The embedded tissues were cut at 70 nm, and the sections were stained with uranyl acetate and lead citrate. Transmission electron microscopy (FEI Tecnai G2 Spirit Bio TWIN, Japan) was used to observe the mitochondrial ultrastructure.

### WGA staining and BODIPY staining

For WGA staining, the paraffin sections were dewaxed, rehydrated, and stained with 5 μg/mL WGA (Thermo Fisher Scientific, cat W11261, USA) in PBS for 30 min at room temperature. Then, the sections were washed with PBS 3 times and observed with a FluoView™ FV3000 confocal microscope (Olympus, Japan).

For BODIPY staining, the mouse hearts were fixed in 4% PFA, dehydrated in 30% sucrose, embedded in OCT and sectioned into 8 μm slices. Frozen sections were thawed at room temperature and stained with 2 μM BODIPY (Thermo Fisher Scientific, cat D3922, USA) for 15–20 min at 37 °C. Then, the sections were washed with PBS 3 times and observed with a FluoView™ FV3000 confocal microscope (Olympus, Japan).

### Immunoblotting and immunoprecipitation

For western blot analysis, mouse hearts or cells were collected and homogenized with RIPA buffer containing protease inhibitor cocktails (Roche, cat 04693132001, Switzerland) and PMSF (1 mM). Then, the lysates were centrifuged at 12,000× *g* for 15 min at 4 °C. The supernatant was collected and boiled with loading buffer for 5 min.

The proteins were separated on an SDS-PAGE gel and transferred to a polyvinylidene difluoride (PVDF) membrane. Then, the membrane was blocked with 5% nonfat milk and incubated with the primary antibody overnight at 4 °C followed by washing with PBST. Then, the membrane was incubated with the indicated HRP-conjugated secondary antibody and detected with a chemiluminescent substrate.

For immunoprecipitation, the mouse hearts were collected and homogenized in IP buffer (25 mM Tris-HCl (pH 7.5), 150 mM NaCl, 2 mM EDTA, 1% Triton X-100, 5% glycerol) containing protease inhibitors cocktails and PMSF (1 mM). Then, the lysates were centrifuged at 12,000× *g* for 15 min at 4 °C. The supernatant was precleared with 1 µg of the appropriate control IgG and 20 µL of Protein A/G PLUS-Agarose (Santa Cruz, cat sc-2003, USA) and centrifuged at 1000× *g* for 5 min at 4 °C. Then, the supernatant was incubated with the indicated primary antibody overnight at 4 °C and incubated with Protein A/G PLUS-Agarose (Santa Cruz, cat sc-2003, USA) for 2–3 h at 4 °C. The immunoprecipitates were centrifuged, washed with IP buffer, and denatured. The proteins were analyzed by immunoblot. The primary antibodies used in the immunoblot analysis and immunoprecipitation are listed in Supplementary Table S5.

### Mitochondrial isolation

Fresh mouse hearts were homogenized in mitochondrial isolation buffer (MIM, 70 mM sucrose, 210 mM mannitol, 5 mM HEPES, 1 mM EGTA, pH 7.2) using a drill-driven Teflon Dounce homogenizer. The homogenate was centrifuged at 800× *g* for 10 min at 4 °C. The supernatant was collected and centrifuged at 800× *g* for 10 min at 4 °C. Then the supernatant was collected and centrifuged at 8000× *g* for 10 min at 4 °C. The light layer was removed, and the pellet of isolated mitochondria was collected and washed three times with MIM. For Ac-coA or β-HB treatment, the mitochondria stored in MIM were treated with Ac-coA or β-HB for 2 h at 37 °C.

### Measurement of OCR and ECAR

Cardiomyocytes were plated in XF24 Cell Culture Microplates before OCR or ECAR detection. Then, an XF24 Extracellular Flux Analyzer (Seahorse Bioscience, USA) was used for the analysis according to the manufacturer’s instructions. The OCR of isolated mitochondria was measured as described previously^52^. Extracellular flux analysis of the metabolic capacity utilizing fatty acids or glucose was performed using an XF Mito Fuel Flex Test Kit (Agilent, cat#103260-100) by detecting the OCR of a specific fuel when other fuel pathways are inhibited according to the manufacturer’s instructions.

### Site-directed mutation of lysine and transfection

In IDH2 and SDHA protein domain, the lysine (K) to arginine (R) mutant plasmids were constructed. Overlap extension PCR with primers bearing mutations was performed to produce the point mutations in *Idh2* and *Sdha*. All mutations were verified by DNA sequencing. The primer sequences are listed in Supplementary Table S4.

### Measurement of the enzyme activity of IDH, SDH, and MDH

Mouse hearts were collected, and enzyme activity was detected immediately. The enzyme activities of IDH and SDH were measured using assay kits purchased from Solarbio (cat BC2160, cat BC0955, China) according to the manufacturer’s instructions. The enzyme activity of MDH was measured using assay kits purchased from Nanjing Jiancheng Bioengineering Institute (cat A021-2-1, China) according to the manufacturer’s instructions.

### Statistical analysis

Quantitation was performed using Image J software. All data are presented as the means ± SEM of at least three independent experiments. The statistical significance of differences between two groups was analyzed by unpaired two-tailed Student’s *t*-test. Differences among multiple groups were analyzed by one-way ANOVA. *P* < 0.05 was considered to indicate statistical significance. *P < 0.05; **P < 0.01; ***P < 0.001.

Further details of materials and methods such as transcriptomic analysis and proteomic analysis are described in Supplementary Data S1.

## Supplementary information

Supplementary informaton

Table S1

Table S2

Table S3

Table S4

Table S5

## Data Availability

We submitted RNA sequencing data to the Gene Expression Omnibus under accession GSE207305 and GSE206797. The authors declare that all relevant data of this study are available within the article or from the corresponding author upon reasonable request.

## References

[1] Piquereau J, Ventura-Clapier R (2018). Maturation of Cardiac Energy Metabolism During Perinatal Development. Front. Physiol..

[2] Porter GA (2011). Bioenergetics, mitochondria, and cardiac myocyte differentiation. Prog. Pediatr. Cardiol..

[3] Bartelds B (1998). Perinatal changes in myocardial supply and flux of fatty acids, carbohydrates, and ketone bodies in lambs. Am. J. Physiol..

[4] Bartelds B (2000). Perinatal changes in myocardial metabolism in lambs. Circulation.

[5] Gong G (2015). Parkin-mediated mitophagy directs perinatal cardiac metabolic maturation in mice. Science.

[6] Breckenridge RA (2013). Hypoxic regulation of hand1 controls the fetal-neonatal switch in cardiac metabolism. PLoS Biol..

[7] Alaynick WA (2007). ERR gamma directs and maintains the transition to oxidative metabolism in the postnatal heart. Cell Metab..

[8] Ali H, Braga L, Giacca M (2020). Cardiac regeneration and remodelling of the cardiomyocyte cytoarchitecture. FEBS J..

[9] Porrello ER (2011). Transient regenerative potential of the neonatal mouse heart. Science.

[10] Puchalska P, Crawford PA (2017). Multi-dimensional Roles of Ketone Bodies in Fuel Metabolism, Signaling, and Therapeutics. Cell Metab..

[11] Ang QY (2020). Ketogenic Diets Alter the Gut Microbiome Resulting in Decreased Intestinal Th17 cells. Cell.

[12] Gors S, Kucia M, Langhammer M, Junghans P, Metges CC (2009). Technical note: Milk composition in mice–methodological aspects and effects of mouse strain and lactation day. J. Dairy Sci..

[13] Laffel L (1999). Ketone bodies: a review of physiology, pathophysiology and application of monitoring to diabetes. Diabetes/Metab. Res. Rev..

[14] Robert C, Watson M (2015). Errors in RNA-Seq quantification affect genes of relevance to human disease. Genome Biol..

[15] Boersma ER, Offringa PJ, Muskiet FA, Chase WM, Simmons IJ (1991). Vitamin E, lipid fractions, and fatty acid composition of colostrum, transitional milk, and mature milk: an international comparative study. Am. J. Clin. Nutr..

[16] Wang WS (2012). Cidea is an essential transcriptional coactivator regulating mammary gland secretion of milk lipids. Nat. Med..

[17] Arima Y (2021). Murine neonatal ketogenesis preserves mitochondrial energetics by preventing protein hyperacetylation. Nat. Metab..

[18] Deng Y (2021). Targeting Mitochondria-Inflammation Circuit by beta-Hydroxybutyrate Mitigates HFpEF. Circ. Res.

[19] Baeza J, Smallegan MJ, Denu JM (2016). Mechanisms and Dynamics of Protein Acetylation in Mitochondria. Trends biochemical Sci..

[20] Xie ZY (2016). Metabolic Regulation of Gene Expression by Histone Lysine beta-Hydroxybutyrylation. Mol. Cell.

[21] Huang H (2021). The regulatory enzymes and protein substrates for the lysine beta-hydroxybutyrylation pathway. Sci. Adv.

[22] Koronowski KB (2021). Ketogenesis impact on liver metabolism revealed by proteomics of lysine beta-hydroxybutyrylation. Cell Rep..

[23] Semple BD, Blomgren K, Gimlin K, Ferriero DM, Noble-Haeusslein LJ (2013). Brain development in rodents and humans: Identifying benchmarks of maturation and vulnerability to injury across species. Prog. Neurobiol..

[24] Maillet M, van Berlo JH, Molkentin JD (2013). Molecular basis of physiological heart growth: fundamental concepts and new players. Nat. Rev. Mol. Cell Biol..

[25] Bruneau BG (2008). The developmental genetics of congenital heart disease. Nature.

[26] Wang PX, Wang JJ, Lei YX, Xiao L, Luo ZC (2012). Impact of Fetal and Infant Exposure to the Chinese Great Famine on the Risk of Hypertension in Adulthood. PLoS One.

[27] Wang NJ (2017). Exposure to severe famine in the prenatal or postnatal period and the development of diabetes in adulthood: an observational study. Diabetologia.

[28] van Abeelen AFM (2012). Cardiovascular consequences of famine in the young. Eur. Heart J..

[29] Girard J, Ferre P, Pegorier JP, Duee PH (1992). Adaptations of Glucose and Fatty-Acid Metabolism during Perinatal-Period and Suckling-Weaning Transition. Physiol. Rev..

[30] Poulsen LL, Siersbk M, Mandrup S (2012). PPARs: Fatty acid sensors controlling metabolism. Semin Cell Dev. Biol..

[31] Grabacka M, Pierzchalska M, Dean M, Reiss K (2016). Regulation of Ketone Body Metabolism and the Role of PPAR alpha. Int. J. Mol. Sci..

[32] Tsuda T, Fitzgerald KK, Temple J (2020). Sudden cardiac death in children and young adults without structural heart disease: a comprehensive review. Rev. Cardiovasc. Med.

[33] Tan JKH, Watanabe T (2018). Determinants of postnatal spleen tissue regeneration and organogenesis. NPJ Regen. Med..

[34] Zhang H (2020). Ketogenesis-generated beta-hydroxybutyrate is an epigenetic regulator of CD8(+) T-cell memory development. Nat. Cell Biol..

[35] Kolwicz SC, Purohit S, Tian R (2013). Cardiac metabolism and its interactions with contraction, growth, and survival of cardiomyocytes. Circ. Res.

[36] Aubert G (2016). The Failing Heart Relies on Ketone Bodies as a Fuel. Circulation.

[37] Heiskanen MA (2015). Different Predictors of Right and Left Ventricular Metabolism in Healthy Middle-Aged Men. Front. Physiol..

[38] Murashige D (2020). Comprehensive quantification of fuel use by the failing and nonfailing human heart. Science.

[39] Monzo L (2021). Myocardial ketone body utilization in patients with heart failure: The impact of oral ketone ester. Metabolism.

[40] Brahma MK, Wende AR, McCommis KS (2021). CrossTalk opposing view: Ketone bodies are not an important metabolic fuel for the heart. J. Physiol.

[41] Newman JC, Verdin E (2014). Ketone bodies as signaling metabolites. Trends Endocrin Met..

[42] Lee T (2019). A Japanese case of mitochondrial 3-hydroxy-3-methylglutaryl-CoA synthase deficiency who presented with severe metabolic acidosis and fatty liver without hypoglycemia. JIMD Rep..

[43] Cardoso AC (2020). Mitochondrial substrate utilization regulates cardiomyocyte cell-cycle progression. Nat. Metab..

[44] Arima Y (2021). Murine neonatal ketogenesis preserves mitochondrial energetics by preventing protein hyperacetylation. Nat. Metab..

[45] Bedi KC (2016). Evidence for Intramyocardial Disruption of Lipid Metabolism and Increased Myocardial Ketone Utilization in Advanced Human Heart Failure. Circulation.

[46] Nielsen R (2019). Cardiovascular Effects of Treatment With the Ketone Body 3-Hydroxybutyrate in Chronic Heart Failure Patients. Circulation.

[47] Wang PP, Tate JM, Lloyd SG (2008). Low carbohydrate diet decreases myocardial insulin signaling and increases susceptibility to myocardial ischemia. Life Sci..

[48] Al-Zaid NS, Dashti HM, Mathew TC, Juggi JS (2007). Low carbohydrate ketogenic diet enhances cardiac tolerance to global ischaemia. Acta Cardiol..

[49] Snorek M (2012). Short-Term Fasting Reduces the Extent of Myocardial Infarction and Incidence of Reperfusion Arrhythmias in Rats. Physiol. Res.

[50] Zou ZT, Sasaguri S, Rajesh KG, Suzuki R (2002). dl-3-Hydroxybutyrate administration prevents myocardial damage after coronary occlusion in rat hearts. Am. J. Physiol. Heart Circ. Physiol.

[51] Bagnall RD (2016). A Prospective Study of Sudden Cardiac Death among Children and Young Adults. N. Engl. J. Med.

[52] Boutagy NE (2015). Isolation of Mitochondria from Minimal Quantities of Mouse Skeletal Muscle for High Throughput Microplate Respiratory Measurements. J. Vis. Exp.

