# Supplementary material for: Neonatal ketone body elevation regulates postnatal heart development by promoting cardiomyocyte mitochondrial maturation and metabolic reprogramming
Source: Cell Discov. 2022 Oct 11;8:106. doi: 10.1038/s41421-022-00447-6 (PMC9553951; doi:10.1038/s41421-022-00447-6)
Supplement: Supplementary file 1 — Supplementary informaton [file 41421_2022_447_MOESM1_ESM.pdf]

Supplementary Data S1. Materials and methods

Transcriptomic analysis

Total RNA was extracted with Trizol™ X-100 (Thermo Fisher Scientific, USA) and a total amount of 2 μg RNA per sample was used as input material for the RNA sample preparations. Sequencing libraries were generated using VAHTS mRNA-seq v2 Library Prep Kit (Illumina, USA). mRNA was purified from total RNA using poly-T oligo-attached magnetic beads and cut into small fragments for cDNA synthesis. After adenylation of 3' ends of DNA fragments, adaptors with hairpin loop structure were ligated, and then PCR was performed. High-throughput RNA sequencing was carried out using the Illumina NovaSeq platform. Raw data were filtered and processed by Q30 and aligned to the mouse reference genome GRCm38/mm10 with Hisat2 software. Quantification of the transcripts was accomplished with featureCounts software guided by the Ensembl gtf gene annotation file (http://www.ensembl.rog/). Fragments Per Kilobase of transcript per Million mapped reads (FPKM) were used for the measurements of the relative abundances of the transcripts. An effective expressed gene required an FPKM score greater than one.

Proteomic analysis

Heart tissues were subjected to protein extraction, digestion and Tandem Mass Tags (TMT) labeling. In brief, tissues were disrupted by tissuelyser and lysed with protein extraction buffer [8 M urea, 75 mM NaCl, 50 mM Tris, pH 8.2, 1% (vol/vol) EDTA-free protease inhibitor, 1 mM NaF, 1 mM β-glycerophosphate, 1 mM sodium orthovanadate, 10 mM sodium pyrophosphate] (Sigma-Aldrich, USA) followed by centrifugation. Protein concentrations were determined using Bradford assay (Beyotime, China). Cysteine residues were reduced with 5 mM DTT (Thermo Fisher Scientific, USA) for 25 min at 56 °C followed by alkylation with 14 mM iodoacetamide (Sigma-Aldrich, USA) for 30 min in dark, and unreacted iodoacetamide was quenched by DTT for 15 min. The urea concentration was diluted with 25 mM Tris (Sigma-Aldrich, pH 8.2, USA) to 1.2 M, and the proteins were digested overnight at 37°C with 5 ng/uL trypsin (Promega, USA, USA) and 1 mM $CaCl_2$ (Sigma-Aldrich, USA). The peptides were desalted by tC18 Sep-Pak column (Waters), followed by TMT 10-Plex (Thermo Fisher Scientific, USA) labeling. After labeling, each of the 3 groups was mixed respectively and desalted by Waters tC18 Sep-Pak column.

For protein quantification, 40 μg TMT-labeled peptide mixture was fractionated using ACQUITY UPLC M-Class with XBridge BEH C18 column (300 μm×150 mm, 1.7μm; 130Å, Waters). 25 fractions were collected by using nonadjacent pooling scheme within a 128 min gradient of 3% buffer B (A: 10 mM ammonium formate, pH 10; B: 100% acetonitrile) for 14 min, 3%–8% buffer B for 1 min, 8%–29% buffer B for 71 min, 29%–41% buffer B for 12 min, 41%–100% buffer B for 1 min, 100% buffer B for 8 min, 100%–3% buffer B for 1 min followed by 20 min in 3% buffer B.

For liquid chromatography-tandem mass spectrometry (LC-MS) analyses, TMT-labeled peptides were resuspended in 0.1% formic acid and analyzed using an LTQ Orbitrap Fusion Lumos mass spectrometer (Thermo Finnigan, USA) coupled to the Easy-nLC 1200. The trap column (75μm×2cm, Acclaim® PepMap100 C18 column, 3μm, 100 Å; DIONEX, Sunnyvale, CA) effluent was transferred to a reverse-phase microcapillary column (75μm×25cm, Acclaim® PepMap RSLC C18 column, 2μm, 100 Å; DIONEX, Sunnyvale, CA). For protein quantification analysis, a 95 min linear gradient (3% to 5% buffer B for 5 s, 5% to 15% buffer B for 40 min, 15% to 28% buffer B for 34.8 min, 28% to 38% buffer B for 12 min, 30% to 100% buffer B for 5 s and 100% buffer B for 8 min) was applied while using the following buffer: 0.1% formic acid (buffer A) and 80% acetonitrile, 0.1% formic acid (buffer B). The Orbitrap

Fusion Lumos mass spectrometer was operated in the data-dependent mode. A full survey scan was obtained for the m/z range of 350–1,500, and the resolution of higher-energy collision dissociation tandem mass spectrometry (HCD MS/MS) was 50,000.

MS raw files (.raw) were searched against the mouse protein sequences obtained from the Universal Protein Resource Database (UniProt, downloaded in July 18, 2018 containing 76,117 sequences) by MaxQuant software [27809316] (version 1.6.5.0) for feature detection, database searching, and protein quantification. N-terminal protein acetylation and methionine oxidation were set as variable modifications, and carbamidomethylation of cysteine residues was set as a fixed modification. The minimum peptide length was 6 amino acids, and the maximum missed cleavage for each peptide was 2. False discovery rates (FDR) cut-offs were set to 0.01 for both proteins and peptides. All other parameters were the default settings of the MaxQuant software.

Metabolomics analysis

For metabolome analysis, the heart samples were mixed and freeze-dried as required. Then the sample was weighted to an EP tube, and extract solution (acetonitrile: methanol: water = 2: 2: 1) containing internal standard (L-2-Chlorophenylalanine, 2 μg/mL) was added. After 30 s vortex, the samples were homogenized at 35 Hz for 4 min and sonicated for 5 min in an ice-water bath. The homogenization and sonication cycle were repeated 3 times. Then the samples were incubated at -40°C for 1 h and centrifuged at 10,000×rpm for 15 min at 4°C. 400 μL of supernatant was transferred to a fresh tube and dried in a vacuum concentrator at 37°C. Then, the dried samples were reconstituted in 200 μL of 50% acetonitrile by sonication on ice for 10 min. The constitution was then centrifuged at 13,000×rpm for 15 min at 4°C, and 75 μL of supernatant was transferred to a fresh glass vial for LC/MS analysis. The quality control sample was prepared by mixing an equal aliquot of the supernatants from all the samples.

The ultra-high-pressure liquid chromatography (UHPLC) separation was carried out using a 1290 Infinity series UHPLC System (Agilent Technologies, USA), equipped with a UPLC BEH Amide column. The mobile phase consisted of 25 mM ammonium acetate and 25 mM ammonia hydroxide in water (pH = 9.75) (A) and acetonitrile (B). The analysis was carried with elution gradient as follows: 0~0.5 min, 95% B; 0.5~7.0 min, 95%~65% B; 7.0~8.0 min, 65%~40% B; 8.0~9.0 min, 40% B; 9.0~9.1 min, 40%~95% B; 9.1~12.0 min, 95% B. The column temperature was 25℃. The auto-sampler temperature was 4°C, and the injection volume was 2 μL (positive) or 2 μL (negative), respectively.

The TripleTOF 6600 mass spectrometry (AB Sciex) was used for its ability to acquire MS/MS spectra on an information-dependent basis (IDA) during an LC/MS experiment. In this mode, the acquisition software (Analyst TF 1.7, AB Sciex) continuously evaluates the full scan survey MS data as it collects and triggers the acquisition of MS/MS spectra depending on preselected criteria. In each cycle, the most intensive 12 precursor ions with intensity above 100 were chosen for MS/MS at collision energy (CE) of 30 eV. The cycle time was 0.56 s. ESI source conditions were set as follows: Gas 1 as 60 psi, Gas 2 as 60 psi, Curtain Gas as 35 psi, Source Temperature as 600°C, Declustering potential as 60 V, Ion Spray Voltage Floating (ISVF) as 5000 V or –4000 V in positive or negative modes, respectively.

MS raw data (.wiff) files were converted to the mzXML format by ProteoWizard, and processed by R package XCMS (version 3.2). The process includes peak deconvolution, alignment and integration. Minfrac and cut off were set as 0.5 and 0.3 respectively. An in-house MS2 database was applied for metabolite identification.

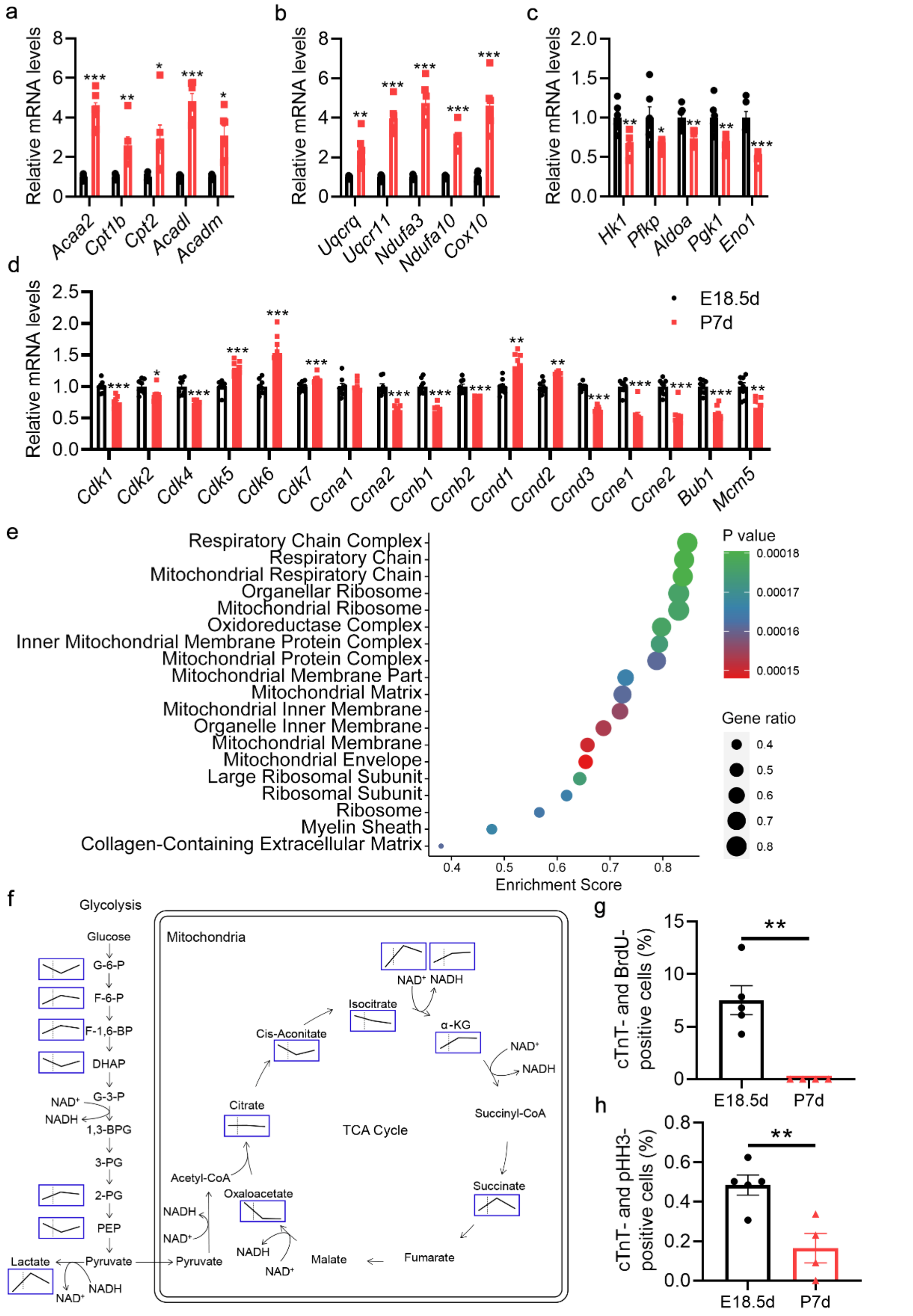

86

Fig. S1 Metabolic reprogramming and cell cycle arrest take place in the neonatal mouse heart. a-d qPCR detection of the mRNA levels of genes related to FA beta oxidation (a, *n* = 6 for each group), mitochondrial oxidative phosphorylation (b, *n* = 6 for each group), glycolysis (c, *n* = 6 for each group) and cell cycle (d, *n* = 8 for each group) in the hearts of E18.5d and P7d mice. **e** GSEA of the proteins in perinatal mouse hearts (P7d vs E18.5d) according to Gene Ontology (GO) cellular component (cc) terms. **f** Metabolome analysis of the glycolytic pathway and the TCA cycle in perinatal mouse hearts

(E18.5d, P1d, and P7d). The dotted line represents the point of birth. **g** The statistics of the percentages of cTnT and BrdU double-positive cells in Fig. 1i (n = 4-5 for each group). **h** The statistics of the percentages of cTnT and pHH3 double-positive cells in Fig. 1j (n = 4-5 for each group). Data are presented as mean ± SEM. and the P values were determined by unpaired two-tailed Student's t-test. *, $P < 0.05$; **, $P < 0.01$; ***, $P < 0.001$.

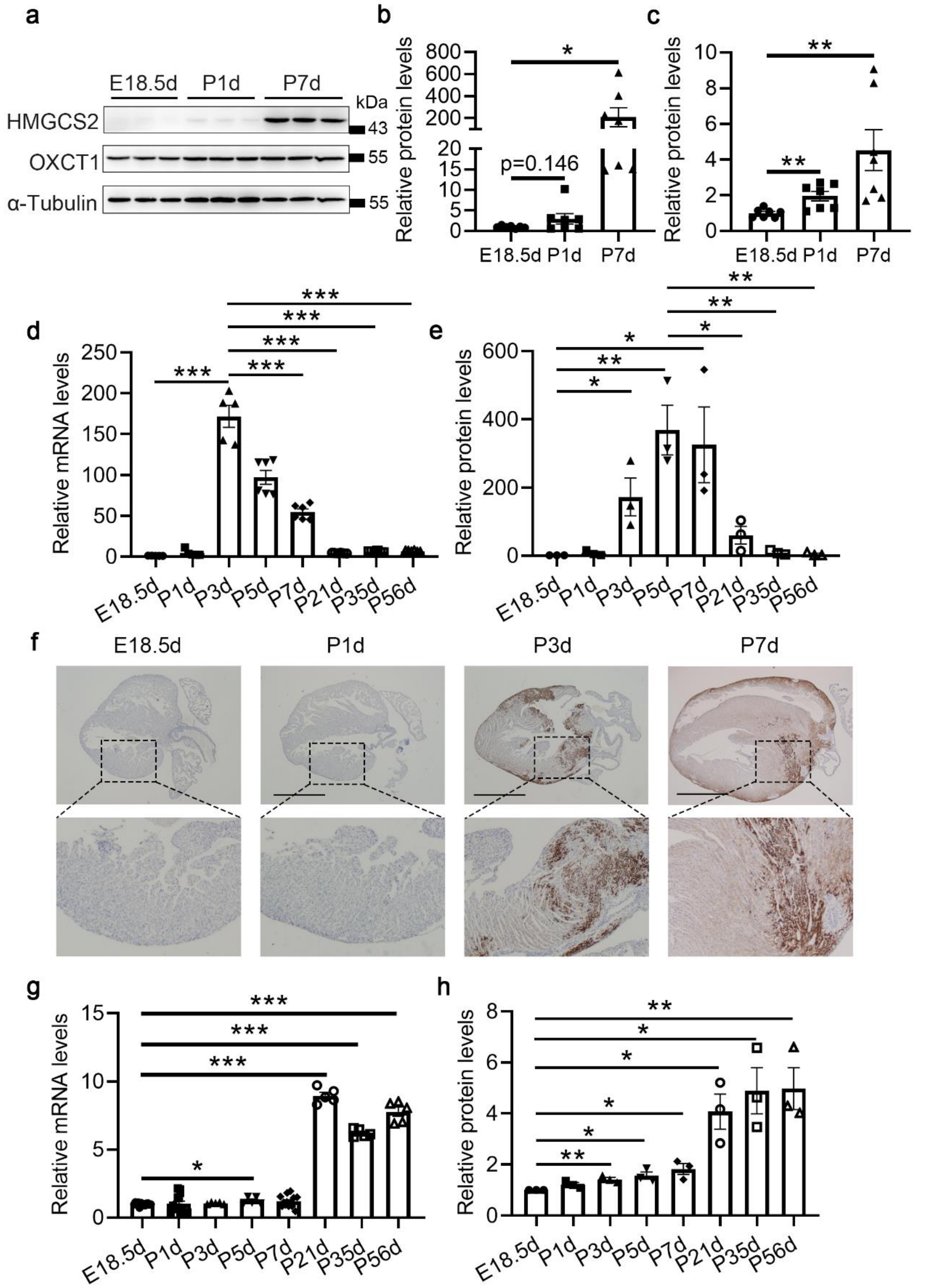

Fig. S2 Enhanced ketogenesis occurs in neonatal mouse heart. **a** Western blot detection of the protein levels of HMGCS2 and OXCT1 in E18.5d, P1d and P7d mouse hearts. **b** Quantitative analysis of the HMGCS2 protein levels in heart tissues in Fig. S2a (*n* = 7 for each group). **c** Quantitative analysis of the OXCT1 protein levels in heart tissues in Fig. S2a (*n* = 7 for each group). **d** qPCR detection of *Hmgcs2* mRNA levels in mouse hearts from the embryonic period to adulthood (*n* = 5-6 for each group).

e Quantitative analysis of the HMGCS2 protein levels in heart tissues in Fig. 2e (*n* = 3 for each group). **f** Immunohistochemical analysis of HMGCS2 expression in the mouse hearts. Scale bar, 2 mm. **g** qPCR detection of *Oxct1* mRNA levels in mouse hearts from the embryonic period to adulthood (*n* = 5-13 for each group). **h** Quantitative analysis of the OXCT1 protein levels in Fig. 2h (*n* = 3 for each group). Data are presented as mean ± SEM. and the *P* values were determined by one-way ANOVA. *, $P < 0.05$; **, $P < 0.01$; ***, $P < 0.001$.

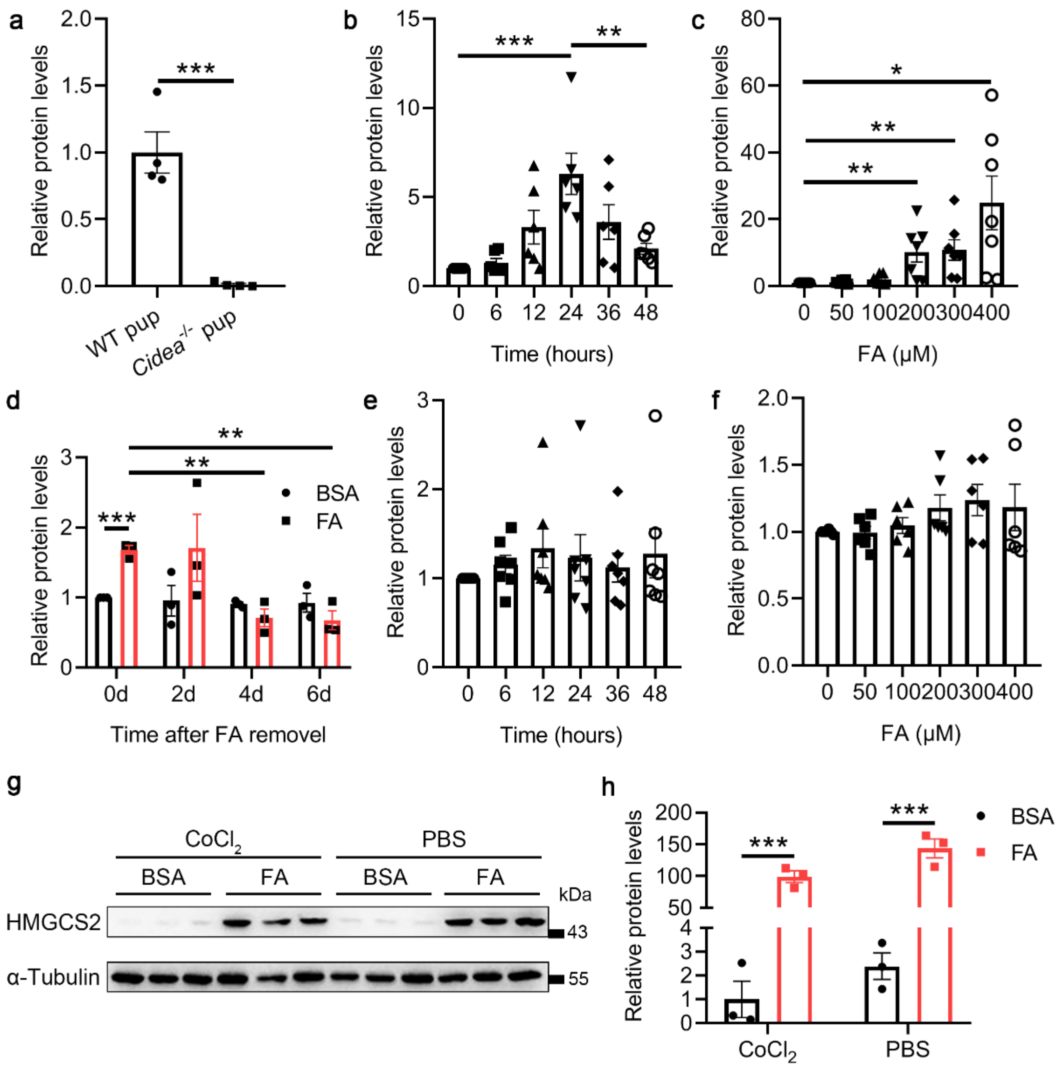

Fig. S3 Colostrum FA promotes transient massive expression of *Hmgcs2* in neonatal mouse heart. **a** Quantitative analysis of the HMGCS2 protein levels in Fig. 3a ($n = 4$ for each group). **b** Quantitative analysis of the HMGCS2 protein levels in Fig. 3c ($n = 6$ for each group). **c** Quantitative analysis of the HMGCS2 protein levels in Fig. 3e ($n = 7$ for each group). **d** Quantitative analysis of the HMGCS2 protein levels in Fig. 3f ($n = 3$ for each group). **e** Quantitative analysis of the OXCT1 protein levels in Fig. 3h ($n = 7$ for each group). **f** Quantitative analysis of the OXCT1 protein levels in Fig. 3j ($n = 6$ for each group). **g** NRVMs were treated with FA (200 μM) and $CoCl_2$ (100 μM) separately or at the same time for 24 h, and then western blotting was performed to detect the protein level of HMGCS2. **h** Quantitative analysis of the HMGCS2 protein levels in Fig. S3g ($n = 3$ for each group). Data are presented as mean ± SEM. and the P values were determined by one-way ANOVA and unpaired two-tailed Student's t-test. *, $P < 0.05$; **, $P < 0.01$; ***, $P < 0.001$.

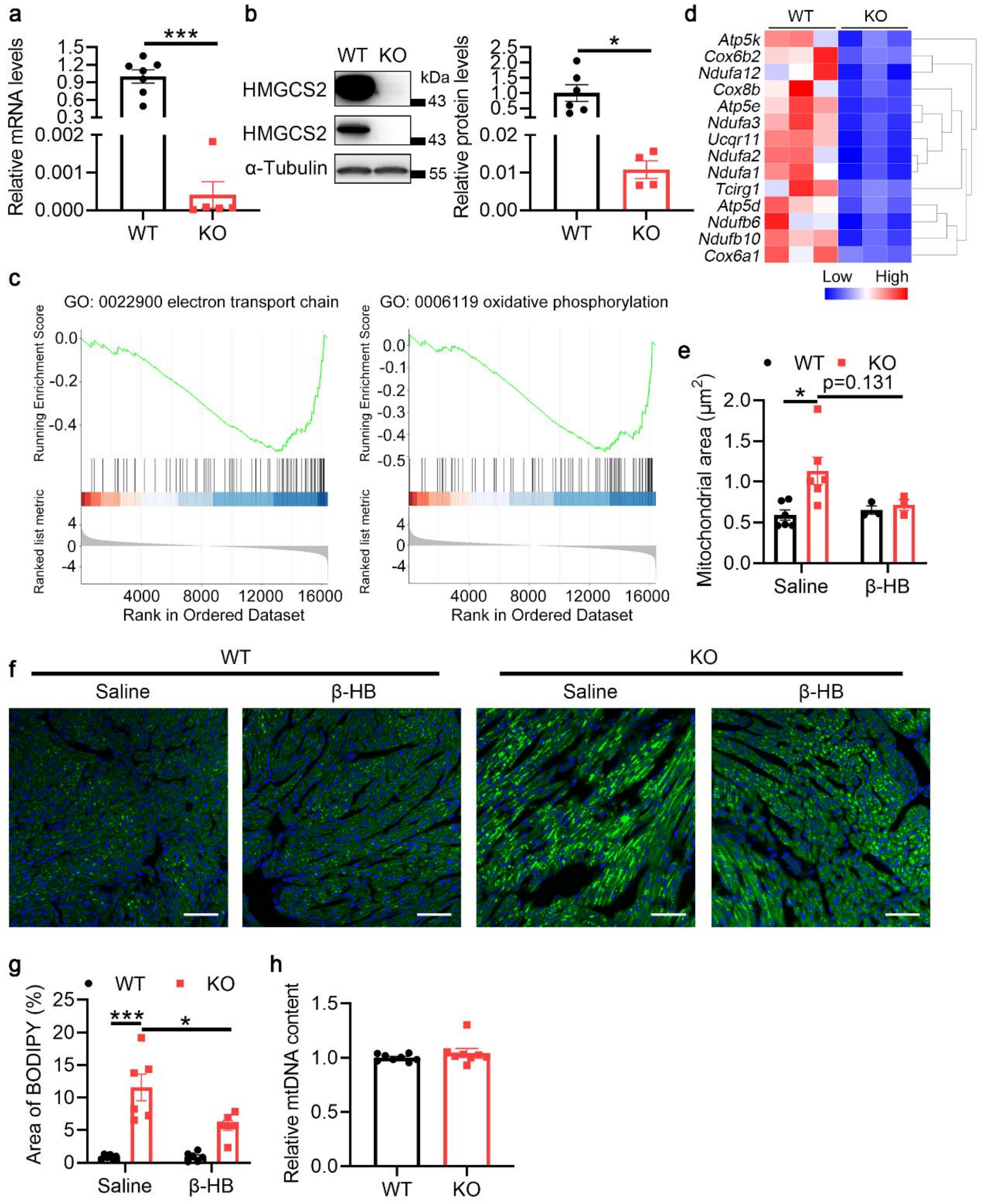

Fig. S4 Ketone body deficiency causes mitochondrial maturation disorder in neonatal mouse heart. **a, b** Detection of the mRNA (a) and protein levels (b) of *Hmgcs2* in the hearts of P5d WT and KO mice. **c** Transcriptomics GSEA score curves: the alteration of mRNA levels related to electron transport chain (left) and oxidative phosphorylation (right) in P3d KO hearts compared with that in WT hearts. **d** Heatmap of mRNA levels related to electron transport chain and oxidative phosphorylation in P3d WT hearts and KO hearts. **e** Statistical analysis of the area of a single mitochondrion in Fig. 4d (n = 3-6 for each group). **f** BODIPY staining in the hearts of P5d WT and KO mice treated with saline or β-HB (400 mg/kg/day though i.p. from P3d to P5d). Scale bar, 50 µm. **g** Statistical analysis of the area of BODIPY in Fig. S4f (n = 6 for each group). **h** qPCR detection of the mtDNA levels (*mt-Co1, mt-Dloop, mt-Atp6*) in the hearts of P5d WT and KO mice (n = 8 for each group). Data are presented as mean ± SEM. and the P values were determined by unpaired two-tailed Student's

t-test. *, $P < 0.05$; ***, $P < 0.001$.

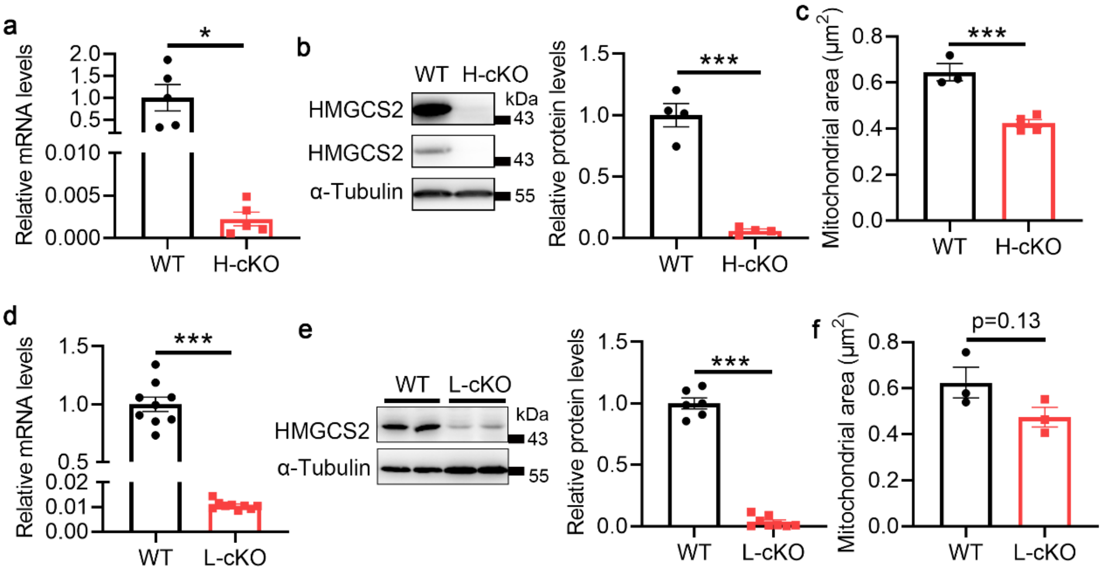

Fig. S5 Heart and liver-specific knockout of *Hmgcs2* has no effect on mitochondrial maturation in neonatal mouse hearts. a, b Detection of the mRNA (n = 5 for each group, a) and protein levels (n = 4 for each group, b) of *Hmgcs2* in the hearts of P5d WT and H-cKO mice. **c** Statistical analysis of the area of a single mitochondrion in Fig. 4i (WT, n = 3; H-cKO, n = 4). **d, e** Detection of the mRNA (n = 9 for each group, d) and protein levels (n = 6-8 for each group, e) of *Hmgcs2* in the livers of P5d WT and L-cKO mice. **f** Statistical analysis of the area of a single mitochondrion in Fig. 4l (n = 3 for each group). Data are presented as mean ± SEM. and the P values were determined by unpaired two-tailed Student's *t*-test. *, $P < 0.05$; ***, $P < 0.001$.

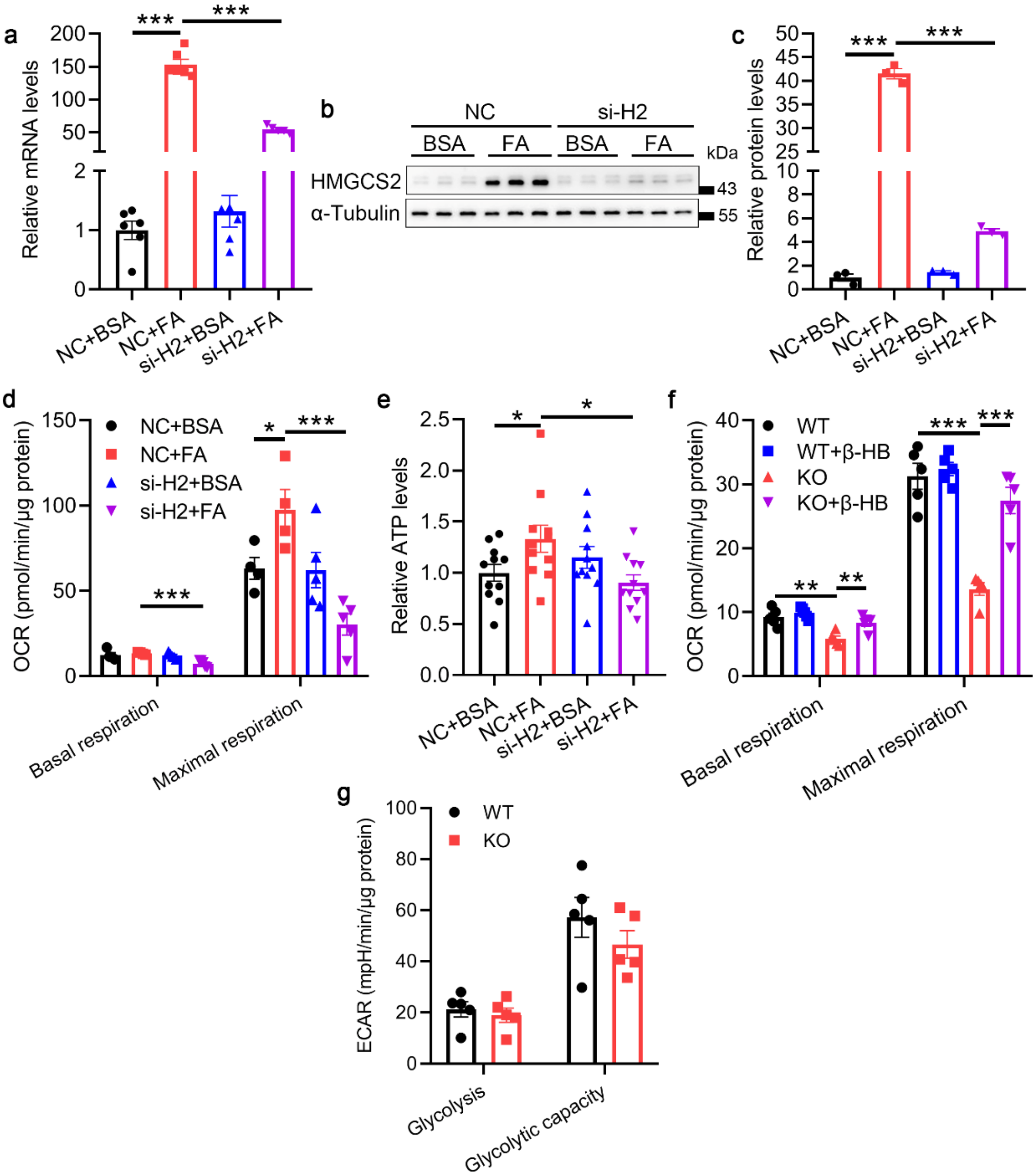

Fig. S6 *Hmgcs2* and ketone body β-HB regulate FA-stimulated mitochondrial oxidative metabolism without affecting glycolysis in neonatal mouse heart. **a** qPCR detection of *Hmgcs2* mRNA levels in negative control and *Hmgcs2*-silenced NRVMs treated with BSA or 200 μM FA (n = 5-6 for each group). **b** Western blot detection of HMGCS2 protein levels in negative control and *Hmgcs2*-silenced NRVMs treated with BSA or 200 μM FA. **c** Quantitative analysis of the HMGCS2 protein levels in Fig. S6b (n = 3 for each group). **d** Statistical analysis of the basal respiration and maximal respiration capacity in Fig. 5c (n = 4-5 for each group). **e** Relative ATP levels in negative control and *Hmgcs2*-silenced NRVMs treated with BSA or 200 μM FA (n = 11 for each group). **f** Statistical analysis of the basal respiration and maximal respiration capacity in Fig. 5d (n = 5 for each group). **g** Statistical analysis of the basal glycolysis and maximal glycolytic capacity in Fig. 5e (n = 5 for each group). Data are presented as mean ± SEM. and the P values were determined by unpaired two-tailed Student's t-test. *, $P < 0.05$; ***, $P < 0.001$.

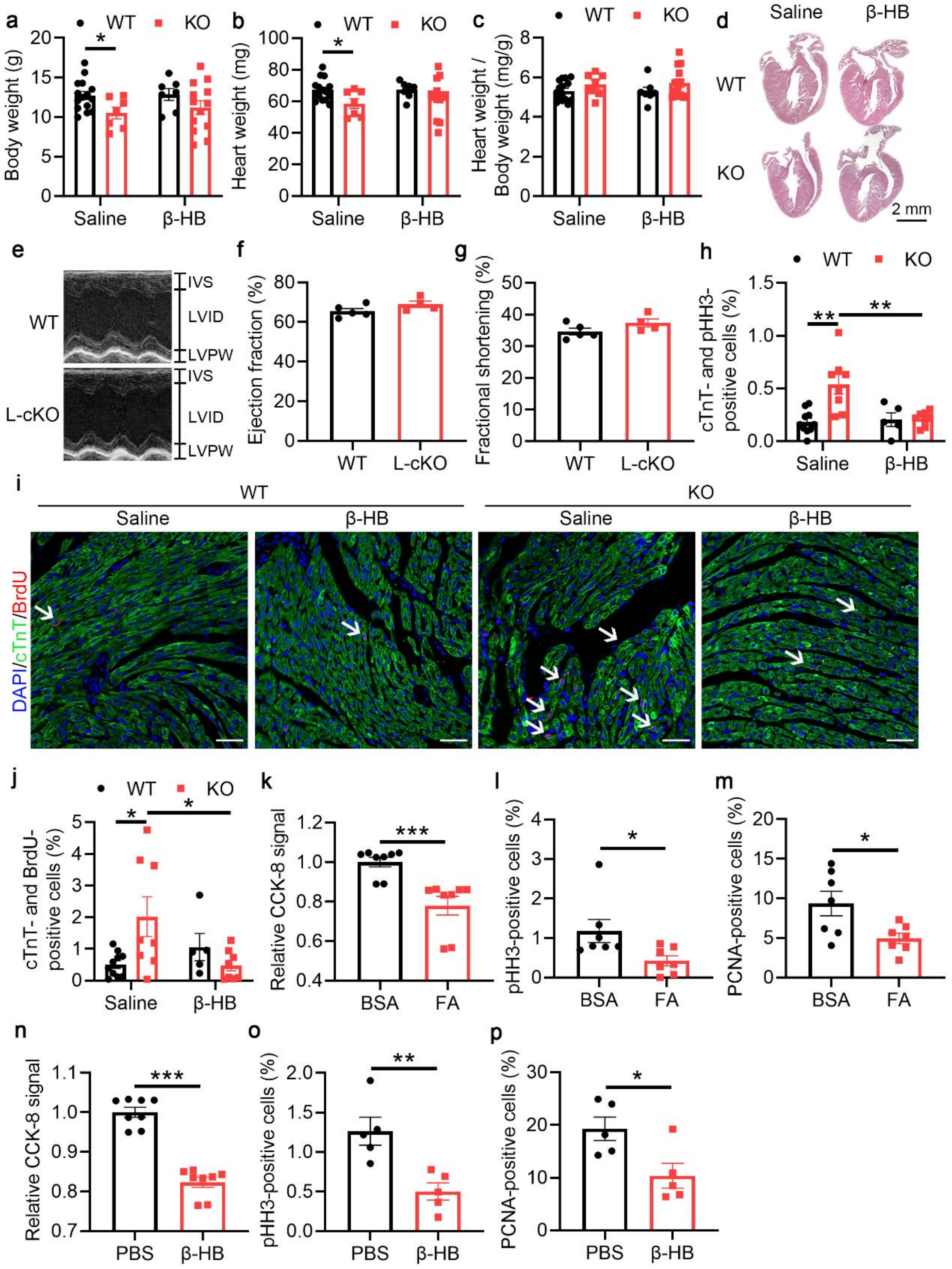

Fig. S7 Neonatal ketone body elevation is essential for postnatal heart development and heart function. **a** Body weights of WT and KO mice at P21d (WT+Saline, *n* = 15; KO+Saline, *n* = 7; WT+β-HB, *n* = 7; KO+β-HB, *n* = 13). **b** Heart weights of WT and KO mice at P21d (WT+Saline, *n* = 15; KO+Saline, *n* = 7; WT+β-HB, *n* = 7; KO+β-HB, *n* = 13). **c** Heart weight/body weight ratios of WT and KO mice at P21d (WT+Saline, *n* = 15; KO+Saline, *n* = 7; WT+β-HB, *n* = 7; KO+β-HB, *n* = 13). **d** Representative longitudinal sections after H&E staining of WT and KO mouse hearts at P21d. Scale bar, 2 mm. **e** Visible images of echocardiographic measurements of WT and L-cKO mice at P21d. **f** Ejection

fractions of WT and L-cKO mice at P21d (*n* = 4-5 for each group). **g** Fractional shortening of WT and L-cKO mice at P21d (*n* = 4-5 for each group). **h** Statistics of the percentages of pHH3-positive cells in Fig. 6g (WT+Saline, *n* = 10; KO+Saline, *n* = 8; WT+β-HB, *n* = 5; KO+β-HB, *n* = 8). **i** Immunofluorescence staining of BrdU and cTnT in the hearts of P5d WT and KO mice treated with saline or β-HB (400 mg/kg/day though i.p. from P3d to P5d). The white arrows indicate cTnT and BrdU double-positive cells. Scale bar, 20 μm. **j** Statistics of the percentages of cTnT and BrdU double-positive cells in Fig. S7i (WT+Saline, *n* = 10; KO+Saline, *n* = 8; WT+β-HB, *n* = 5; KO+β-HB, *n* = 8). **k** CCK-8 assay of H9C2 cells treated with BSA or 200 μM FA (*n* = 8 for each group). **l** Statistics of the percentages of pHH3-positive cells in Fig. 6h (*n* = 7 for each group). **m** Statistics of the percentages of PCNA-positive cells in Fig. 6h (*n* = 7 for each group). **n** CCK-8 assay of H9C2 cells treated with PBS or 5 mM β-HB (*n* = 8 for each group). **o** Statistics of the percentages of pHH3-positive cells in Fig. 6i (*n* = 5 for each group). **p** Statistics of the percentages of PCNA-positive cells in Fig. 6i (*n* = 5 for each group). Data are presented as mean ± SEM. and the *P* values were determined by unpaired two-tailed Student's *t*-test. *, $P < 0.05$; **, $P < 0.01$; ***, $P < 0.001$.

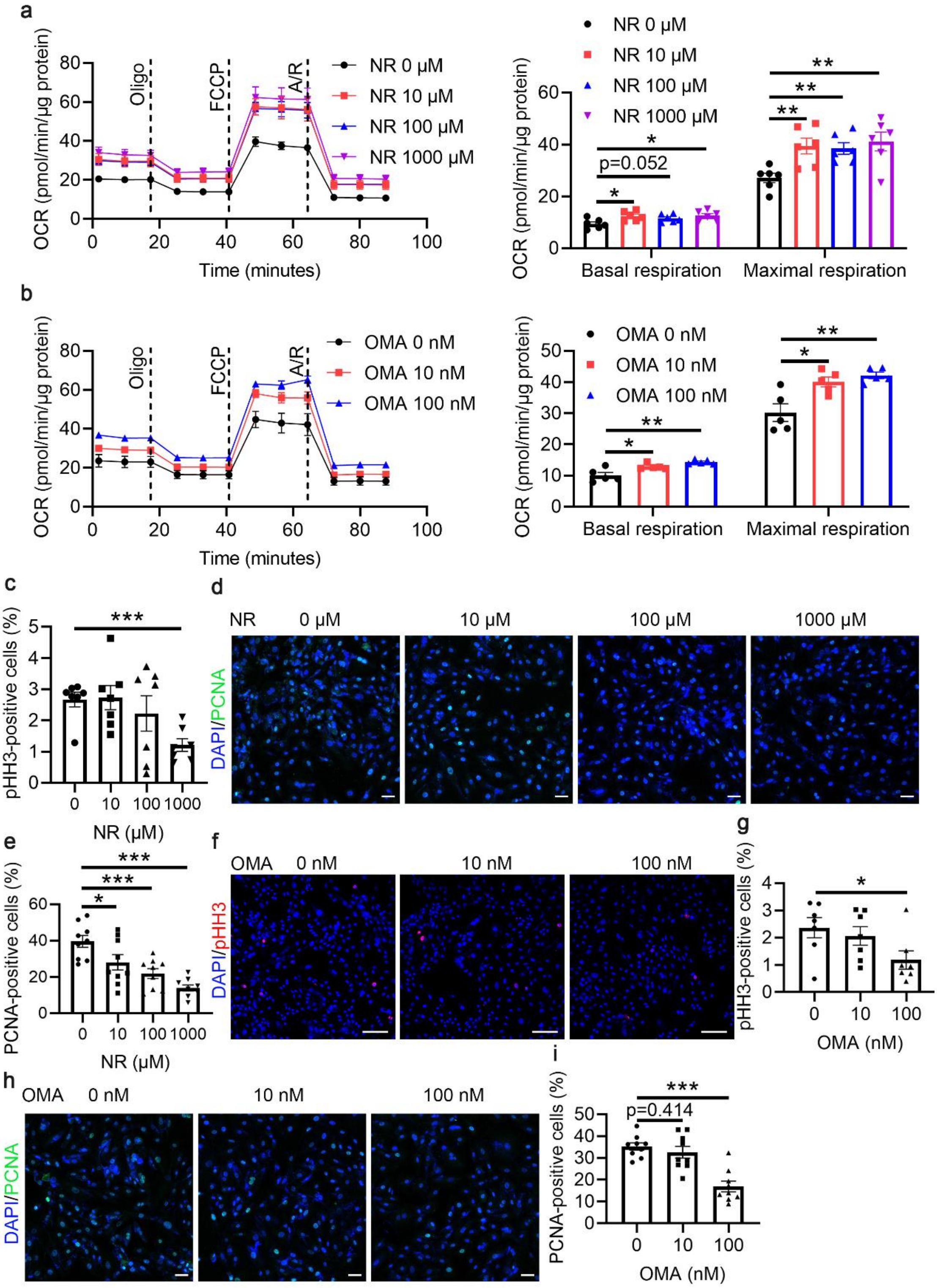

Fig. S8 Enhancing mitochondrial function inhibits cardiomyocyte cell proliferation. **a** Extracellular metabolic flux analysis of the mitochondrial OCR in H9C2 cells treated with NR at different concentrations for 24h and the statistical analysis of the basal respiration and maximal respiration capacity (*n* = 6 for each group). **b** Extracellular metabolic flux analysis of the mitochondrial OCR in H9C2 cells treated with OMA at different concentrations for 24h and the statistical analysis of the basal respiration and maximal respiration capacity (*n* = 5 for each group). **c** Statistics of the percentages of

pHH3-positive cells in Fig. 6j (n = 7 for each group). **d** Immunofluorescence staining of PCNA in H9C2 cells treated with DMSO or NR for 24h. Scale bar, 50 μm. **e** Statistics of the percentages of PCNA-positive cells in Fig. S8d (n = 9 for each group). **f** Immunofluorescence staining of pHH3 in H9C2 cells treated with ddH_2O or OMA for 24h. Scale bar, 100 μm. **g** Statistics of the percentages of pHH3-positive cells in Fig. S8f (n = 7 for each group). **h** Immunofluorescence staining of PCNA in H9C2 cells treated with ddH_2O or OMA for 24h. Scale bar, 50 μm. **i** Statistics of the percentages of PCNA-positive cells in Fig. S8h (n = 9 for each group). Data are presented as mean ± SEM. and the P values were determined by one-way ANOVA and unpaired two-tailed Student's t-test. *, $P < 0.05$; **, $P < 0.01$; ***, $P < 0.001$.

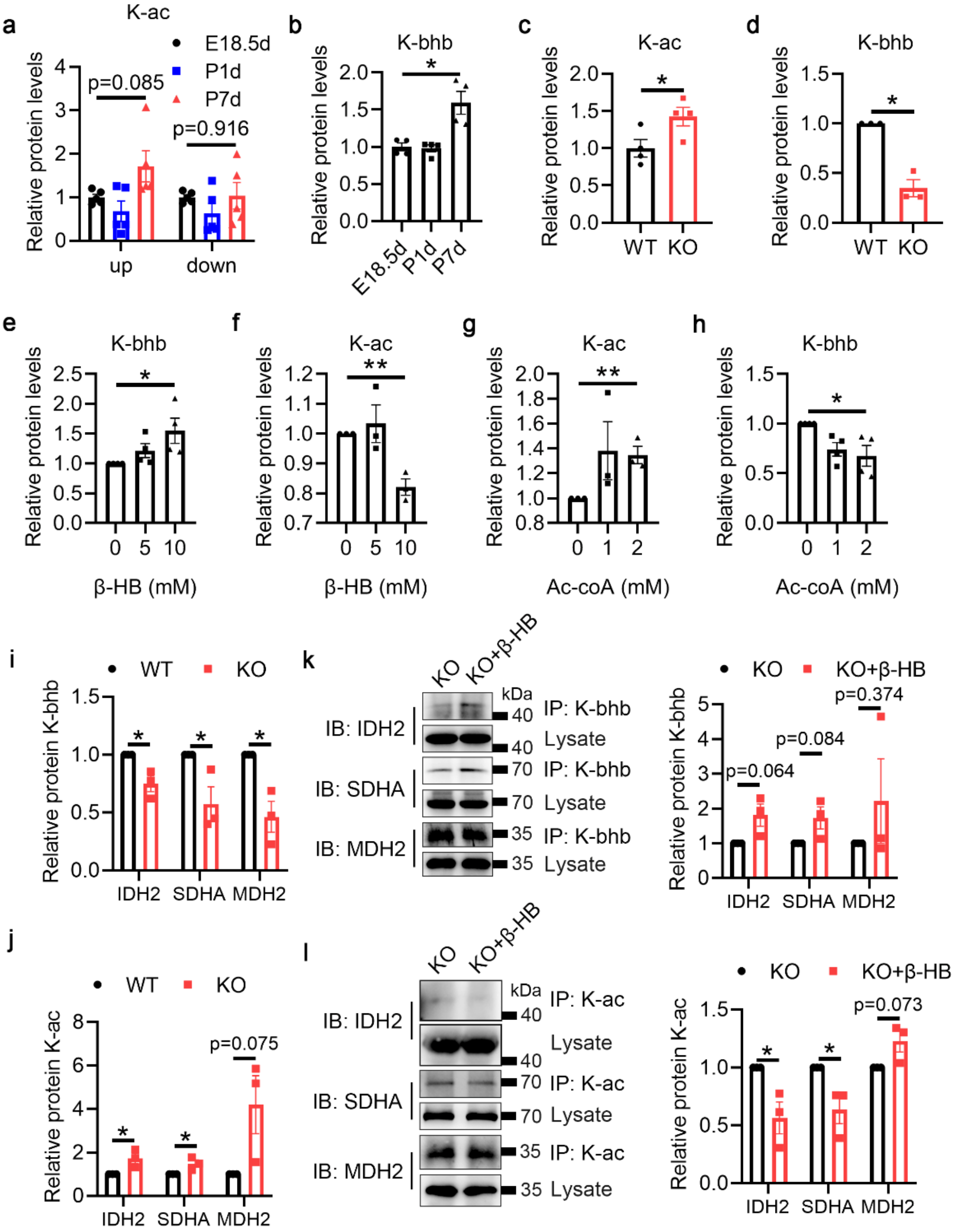

Fig. S9 Mitochondrial protein β-hydroxybutyrylation and acetylation may account for the regulation of mitochondrial maturation by ketone body β-HB. **a** Statistics of the mitochondrial protein K-ac in Fig. 7a (n = 5 for each group). Up: Grayscale analysis of the bands in the dashed frame in the upper part. Down: Grayscale analysis of the bands in the dashed box in the lower part. **b** Statistics of the mitochondrial protein K-bhb in Fig. 7b (n = 4 for each group). **c** Statistics of the mitochondrial protein K-ac in Fig. 7c (n = 4 for each group). **d** Statistics of the mitochondrial protein K-bhb in Fig. 7d (n = 3 for each group). **e** Statistics of the mitochondrial protein K-bhb in Fig. 7f (n = 4 for each group). **f** Statistics of the mitochondrial protein K-ac in Fig. 7f (n = 3 for each group). **g** Statistics of the mitochondrial protein K-ac in Fig. 7g (n = 3 for each group). **h** Statistics of the mitochondrial protein K-bhb in Fig. 7g (n = 4 for each group). **i** Statistics of the protein K-bhb of IDH2, SDHA and MDH2 in Fig. 7h (n = 3 for each group). **j** Statistics of the protein K-ac of IDH2, SDHA and MDH2 in Fig. 7i (n = 3

for each group). **k** Immunoprecipitation assays on the hearts of KO mice at P5d treated with saline or β-HB (400 mg/kg/day though i.p. from P3d to P5d). IP: Anti-K-bhb. IB: IDH2, SDHA and MDH2. **l** Immunoprecipitation assays on the hearts of KO mice at P5d treated with saline or β-HB (400 mg/kg/day though i.p. from P3d to P5d). IP: Anti-K-ac. IB: IDH2, SDHA and MDH2. Data are presented as mean ± SEM. and the *P* values were determined by one-way ANOVA and unpaired two-tailed Student's *t*-test. *, $P < 0.05$; **, $P < 0.01$.

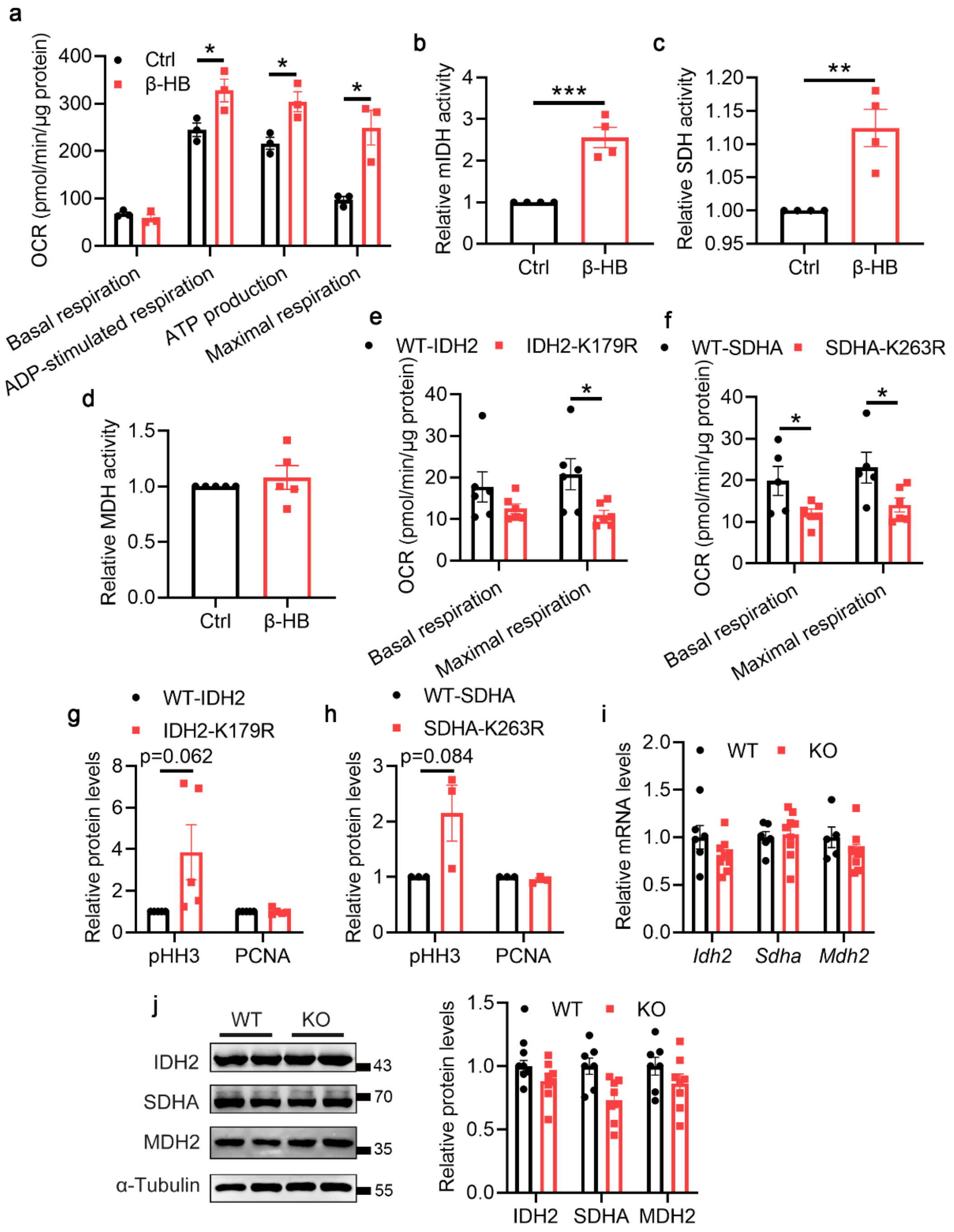

Fig. S10 Ketone body β-HB promotes the enzyme activity of IDH2 and SDHA. **a** Statistical analysis of the OCR in Fig. 7m (*n* = 3 for each group). **b** Relative mitochondrial IDH (mIDH) activity of 293T cells treated with PBS (Control, Ctrl) or 5 mM β-HB (*n* = 4 for each group). **c** Relative SDH activity of 293T cells treated with PBS or 5 mM β-HB (*n* = 4 for each group). **d** Relative MDH activity of 293T cells treated with PBS or 5 mM β-HB (*n* = 5 for each group). **e** Statistical analysis of the OCR in Fig. 7p (*n* = 6 for each group). **f** Statistical analysis of the OCR in Fig. 7q (WT-SDHA, *n* = 5; SDHA-K263R, *n* = 6). **g-h** Statistical analysis of protein levels of pHH3 and PCNA in Fig. 7r (*n* = 3-5 for each group). **i** qPCR detection of the mRNA of *Idh2*, *Sdha* and *Mdh2* in the hearts of P5d WT and KO mice (*n* = 5-9). **j** Western blot detection of the protein levels of IDH2, SDHA and MDH2 in the hearts of P5d WT and KO mice and the statistical analysis of the protein levels of IDH2, SDHA and MDH2 (*n* = 7-8). Data are presented as mean ± SEM. and the P values were determined by unpaired two-tailed Student's t-test. *, $P < 0.05$; **, $P < 0.01$; ***, $P < 0.001$.

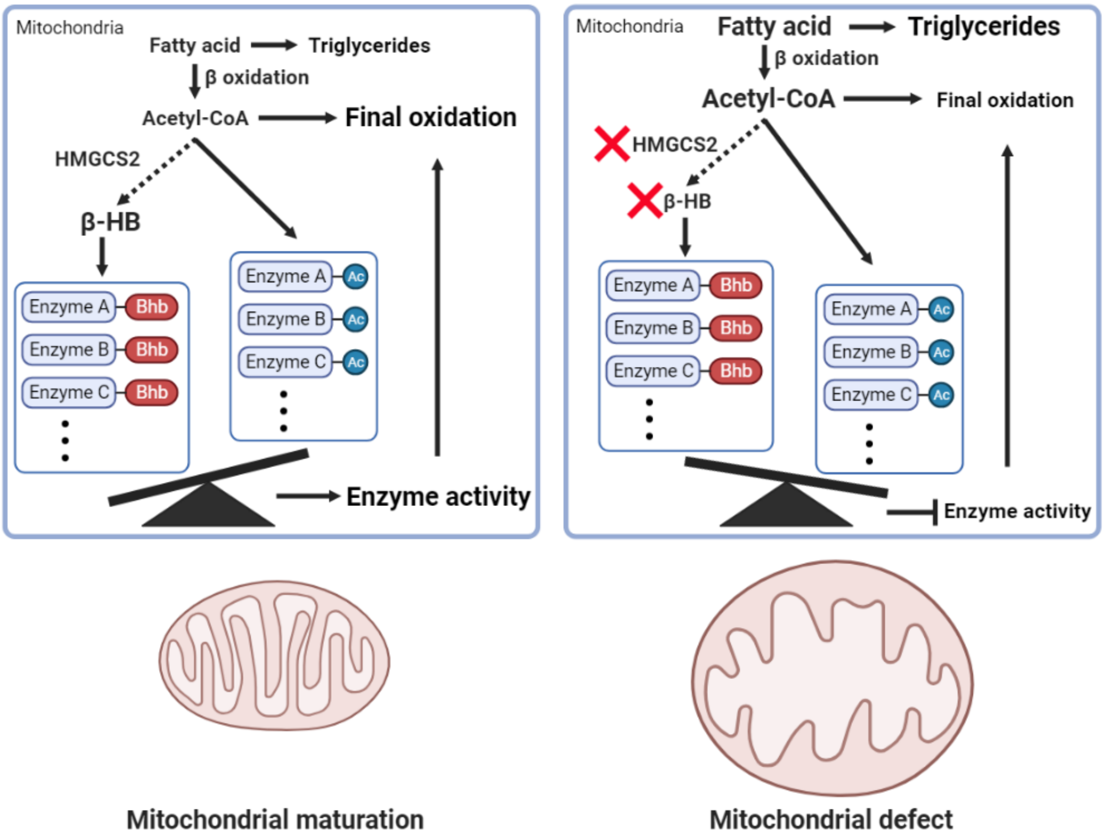

Fig. S11 Proposed model of ketone body regulating mitochondrial maturation in the postnatal heart. The FA in the milk sucked by newborns are absorbed by cardiomyocytes and enter three metabolic pathways: mitochondrial oxidation, triglyceride synthesis, and ketogenesis. Fatty acids stimulate the expression of *Hmgcs2* in the heart. β-HB can participate in the K-bhb of metabolic enzymes (such as IDH2 and SDHA) in mitochondria, which is essential for the activity of these enzymes and mitochondrial function (left). After *Hmgcs2* is deleted, the K-bhb of metabolic enzymes in mitochondria is decreased. In addition, due to the lack of ketogenesis, acetyl-CoA is accumulated and participates in the K-ac of metabolic enzymes in mitochondria. The decrease of K-bhb and the increase of K-ac together lead to the decrease of enzyme activity and ultimately cause the abnormality of mitochondrial function (right).
